# A competence of embryo-derived tissues of tetraploid cultivated wheat species *Triticum dicoccum* and *Triticum timopheevii* for efficient and stable transgenesis mediated by particle inflow gun

**DOI:** 10.1186/s12870-020-02580-4

**Published:** 2020-10-14

**Authors:** Dmitry Miroshnichenko, Anna Klementyeva, Alexander Pushin, Sergey Dolgov

**Affiliations:** 1 Branch of Shemyakin and Ovchinnikov Institute of Bioorganic Chemistry RAS, Pushchino, Moscow Region 142290 Russian Federation; 2 All-Russia Research Institute of Agricultural Biotechnology, Moscow, 127550 Russian Federation; 3 Kurchatov Genomics Center—ARRIAB, All-Russia Research Institute of Agricultural Biotechnology, Moscow, 127550 Russian Federation

**Keywords:** Tetraploid wheat, Particle bombardment, Immature embryos, Mature embryos, Callus type, Multiple insertions, Transgene inheritance

## Abstract

**Background:**

The ability to engineer cereal crops by gene transfer technology is a powerful and informative tool for discovering and studying functions of genes controlling environmental adaptability and nutritional value. Tetraploid wheat species such as emmer wheat and Timopheevi wheat are the oldest cereal crops cultivated in various world areas long before the Christian era. Nowadays, these hulled wheat species are gaining new interest as donors for gene pools responsible for the improved grain yield and quality, tolerance for abiotic and biotic stress, resistance to pests and disease. The establishing of efficient gene transfer techniques for emmer and Timopheevi wheat may help in creation of modern polyploid wheat varieties.

**Results:**

In the present study, we describe a robust protocol for the production of fertile transgenic plants of cultivated emmer wheat (Russian cv. ‘Runo’) using a biolistic delivery of a plasmid encoding the gene of green fluorescent protein (GFP) and an herbicide resistance gene (*BAR*). Both the origin of target tissues (mature or immature embryos) and the type of morphogenic calli (white or translucent) influenced the efficiency of stable transgenic plant production in emmer wheat. The bombardment of nodular white compact calluses is a major factor allowed to achieve the highest transformation efficiency of emmer wheat (on average, 12.9%) confirmed by fluorescence, PCR, and Southern blot. In the absence of donor plants for isolation of immature embryos, mature embryo-derived calluses could be used as alternative tissues for recovering transgenic emmer plants with a frequency of 2.1%. The biolistic procedure based on the bombardment of immature embryo-derived calluses was also successful for the generation of transgenic *Triticum timopheevii* wheat plants (transformation efficiency of 0.5%). Most of the primary events transmitted the transgene expression to the sexual progeny.

**Conclusion:**

The procedures described here can be further used to study the functional biology and contribute to the agronomic improvement of wheat. We also recommend involving in such research the Russian emmer wheat cv. ‘Runo’, which demonstrates a high capacity for biolistic-mediated transformation, exceeding the previously reported values for different genotypes of polyploid wheat.

## Background

Wheat (*Triticum* L.) is the major cereal crop in a temperate climate and, along with rice and corn, it plays a central role in the global food supply, providing a human diet with an abundance of calories and protein. An important group within the genus *Triticum* is the tetraploid wheat species (2n = 4x = 28), which phylogenetically can be divided into two sub-groups [1]. One sub-group consists of the species with AABB genome, which includes the durum wheat, the only tetraploid species actively cultivated in various countries for a macaroni and pasta production; another sub-group consists of the forms with AAGG genome. Despite the centrality of hexaploid bread wheat (*T.aestivum* L., 2n = 6x = 42) in the actual world production, the current wheat breeding programs increasingly involve the various ancient tetraploid wild and cultivated species for reinforcement of numerous economically important traits such as yield, grain constituents, resistance to diseases and pests, and tolerance to various abiotic stresses [2, 3].

The tetraploid emmer wheat (*T. dicoccon* [syn. *T. turgidum* L. subsp. *dicoccon* (Schrank) Thell.]) is one of the oldest cereal crops [4]. As the direct progenitor for the modern cultivated wheat, emmer wheat has the same genomic formula (AABB) as durum wheat (*T. durum* Desf. [syn *T. turgidum* ssp. *durum* (Desf.) Husn.]) and contributed two genomes to bread wheat (AABBDD) [1]. Because of the 10,000-year history of cultivation, the intraspecific classification of emmer wheat is quite complicated, so it is generally divided into wild emmer (*T. dicoccoides* (Korn. Körn ex Asch. et Graebn.) Schweinf.) and cultivated emmer (*T. dicoccum* (Schrank) Schuebl.) [4]. In contrast to emmer wheat, tetraploid species with AAGG genome, such as the domesticated Timopheevi wheat (*T. timopheevii* (Zhuk.) [syn *T.timopheevii* ssp. *Timopheevii*]) and its wild progenitor *T. araraticum* Jakubz. (syn. *T.timopheevii* ssp. *araraticum*) are no longer cultivated [5]. Both emmer and Timopheevi wheat are self-pollinated species that are characterized by persistent enclosing hulls. In recent decades, these species have attracted considerable attention of breeders as a natural source of the gene pool for the production of new immune varieties of wheat [2, 3, 6, 7]. They also used in various studies as models for studying the evolution and polyploidy of the wheat genome [3, 8, 9]*.*

Various biotechnological techniques could be applied for the acceleration of the wheat breeding process. Among those techniques, marker-assistant selection, DNA mapping, genomic selection, genome editing and genetic engineering are currently explored in advanced crop breeding programs [10]. Genetic transformation, which gives a chance to introduce specific genes that encode important beneficial traits, is an effective mean for improvement of existing elite varieties [11]. The ability to engineer transgenic plants is also a powerful and informative tool for studying functions and regulations of genes controlling important traits, especially since the most of genetic resources in ancient tetraploid wheats are still not identified. Despite the obvious potential, emmer wheat and Timopheevi wheat have not received considerable attention for genetic transformation. To date, only two reports describing the generation of transgenic plants of the Indian emmer wheat DDK1001 (*T. dicoccum*) by biolistic and agrobacterium-mediated gene transfer techniques were published [12, 13]. Although *BAR*, *HPT* and *GUS* genes were successfully transferred and expressed in transgenic emmer plants, several shortcomings were inherent for the proposed transformation protocols. Owning to the poor growth of T_0_ transformants, a significant part of in vitro regenerated plants died during the adaptation to the glasshouse. Moreover, most of the survived plants failed to produce seeds; while the seed quality of the rest of transgenic plants was not satisfactory. For this reason, no information concerning the inheritance of the introduced genes is available for emmer wheat DDK1001 [12, 13]. Recently transgenic plants of wild emmer wheat MG4343 were produced by Italian researchers with a frequency of 1.76%, and the successful inheritance of the introduced *Tapgip1* gene to the next generations was confirmed [14].

The restricted number of studies on emmer wheat and the absence of studies concerned to the genetic transformation of Timopheevi wheat have motivated us to develop a biolistic-mediated method for introducing alien genes into their genome using various types of embryo-derived tissues. Since the emmer wheat is regaining popularity in Russia, several promising genotypes are currently under the commercialization, from which the variety ‘Runo’ is the first modern commercial variety of emmer wheat in Russia. Selected by the breeders from the National Center of Grain named after P.P. Lukyanenko and All-Russian Institute of Plant Genetic Resources, ‘Runo’ is approved for cultivation in Krasnodar and Rostov areas for feed and food since 2009 [15].

Currently, the overwhelming majority of protocols relays on the application of immature zygotic embryos (IE) as the primary tissue source for the regeneration of transgenic wheat plant [11]. The continuous growth of donor plants in a glasshouse is required to ensure efficient transformation since the proper physiological stage of IE capable of developing morphogenic callus is strictly limited to one or two days. Mature embryos (ME) are now increasingly recognized as a low-cost and time-saving alternative target tissue for DNA transfer, which could be used year-round without restrictions [16, 17]. In wheat, however, the recalcitrancy of ME in tissue culture limits the application of this explant type for genetic transformation [18]. Our previous study revealed that emmer wheat ‘Runo’ displayed high plant regeneration ability when the IE tissues used as initial explants [19]. In ME-derived culture, it exhibits a better level of morphogenic callus formation than many other polyploid wheat species and cultivars [19]. Since *T. timopheevii* showed a much lower ability to generate plants in vitro [19], we have performed a series of experiments to improve its ability to produce plants; however, the acceptable level of morphogenesis still was not achieved [20]. Recently we have succeeded to notably increase the efficiency of IE-derived tissue of recalcitrant wheat species, such as diploid einkorn (*T. monococcum* L.), primarily by the incorporation of Daminozide in the induction medium [21]. This strategy was also used here to stimulate the morphogenic response in IE of Timopheevi wheat before delivering foreign genes.

In the method described below, transgenic plants of emmer wheat ‘Runo’ may be routinely obtained from the bombarded explants by the proper choice of competent IE-and seed-derived calli. The use of morphogenic IE explants also allowed for the first time to produce transgenic plants of Timopheevi wheat.

## Results

### Generation of transgenic plants of emmer wheat

#### IE-derived tissues

**Fig. 1:**
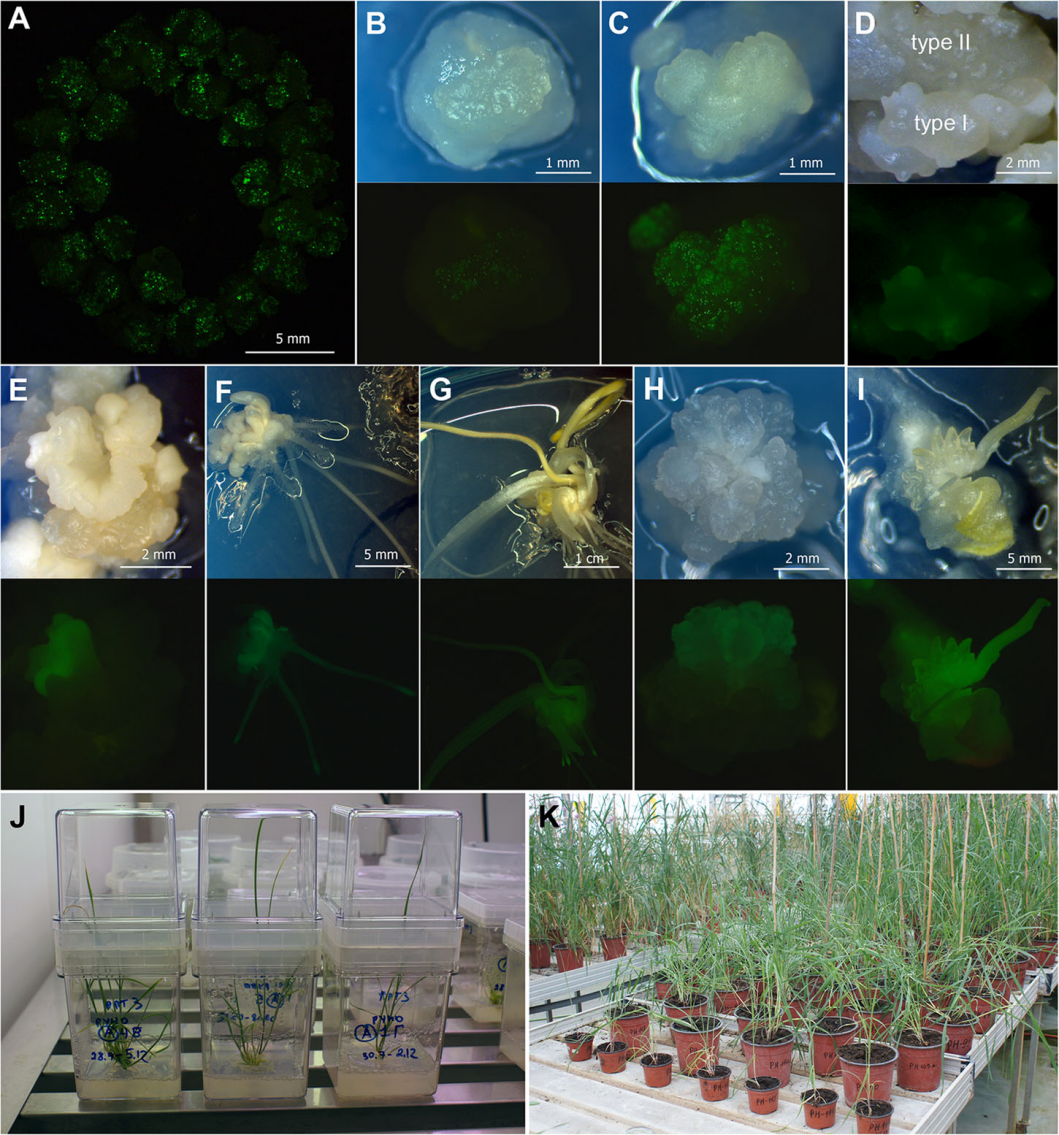
Production of transgenic plants of emmer wheat ‘Runo’ (*T. dicoccum* (Schrank.)). **a,b,c,d.** Transient *GFP* gene expression; morphogenic explants arranged in a circle on the plate with the osmotic medium for bombardment, 16 h after plasmid delivery (**a**); effect of callus type on the transient *GFP* expression, type I (**b**) and type II (**c**) callus, 20 h after bombardment; mixing explant with type I and type II callus (**d**), 21 days after bombardment. **e.** GFP-positive transgenic morphogenic cluster, 50 days of culture. **f.** Formation of GFP-positive transgenic single embryo-like structure with roots, 65 days of culture. **g.** Development of transgenic regenerating plant, 80 days of culture. **h.** Extended morphogenic areas with a bright GFP fluorescence, 60 days of culture. **i**. Development of multiple GFP-positive plantlets, 75 days of culture. **j**. in vitro rooting and development of putative transgenic plants in Magenta boxes. **k**. Putative transgenic plants of emmer wheat in the greenhouse, different stages of plant development. Tissues were photographed under white light or UV light using the GFP filter set (EX BP 470/40, BS FT 495, EM LP 550)

Previous experiments [19] revealed that isolated immature embryos of tetraploid emmer wheat cv. ‘Runo’ produce two types of regenerable embryogenic/organogenic calluses. Type I, representing white nodular organogenic structures (Fig. 1c), mostly arises on the edges of cultured embryos starting from the 5–7 days of initiation. Type II appears on the 10–15 days of culture and it is characterized by the softer translucent nodular callus (Fig. 1b). In several independent experimental sets, each including from 50 to 150 explants, a total of 653 embryogenic/organogenic pieces of Type 1 callus and 476 pieces of Type II calluses were bombarded with the psGFP-BAR. After the plasmid delivery, both types of explants began to display transient *GFP* expression within 24 h (Fig. 1a). However, the intensity and the number of GFP-positive spots were generally higher when the Type I was used (Fig. 1b,c). After two-three weeks of selection, Type II continued to show lower GFP fluorescence intensity, while significant areas of Type I structures were covered with green spots (Fig. 1d). Every next subculture the number of fluorescing spots declined in both types of explants. Some of the sectors, however, remained brightly fluorescent producing the GFP-positive transgenic morphogenic clusters (Fig. 1e). Such clusters, separated and transferred to fresh medium, were able to form a  single embryo-like structure (Fig. 1f), which later developed into the whole plantlets expressing *GFP* (Fig. 1g). In some cases, extended morphogenic areas with bright GFP fluorescence were arising (Fig. 1h). After the transferring to DM, the only such cluster could produce a series of transgenic somatic embryos (Fig. 1i), which subsequently easily turned into two-to-five transgenic plants on RM. Since these plantlets originated from the same explant, they were counted as the one event.

The vast majority of shoots selected after visual GFP selection vigorously grew and readily rooted in the presence of PPT (Fig. 1j), and then quickly adapted to greenhouse conditions (Fig. 1k). None of the transferred plantlets died during the acclimatization. A total of 121 putative transgenic plants were established in the greenhouse. All of them were subjected for the PCR and RT-PCR analysis (Additional file 1: Figure S1), which confirmed the presence of * GFP* and *BAR* sequences in 115 primary plants, indicating a low portion of ‘escapes’ (less than 5%). The type of the IE derived embryogenic/organogenic explants subjected to bombardment significantly influenced the success of the genetic transformation of emmer wheat. The delivery of transgenes into the nodular translucent callus (Type II) allowed producing independent events with an average frequency of 3.4% (Table 1). The use of the Type I explants (nodular white compact) significantly increased the transformation efficiency. On average, 12.9% of the bombarded Type I explants gave rise to transgenic events, while the transformation itself was more stable since the production of transgenic plants ranged within 6.0–19.0% in independent experiments (Table 1).

**Table 1:** Efficiency of transgenic plant production after biolistic- mediated transformation of immature embryo-derived tissues of emmer wheat ‘Runo’ (*T.dicoccum* (Schrank))

Morphogenic tissue type^a^	Experiment No.	No. independent explants bombarded	No. independent explants produced plantlets after dual GFP + PPT selection	No. putative transgenic plants selected in vitro		No. independent explants produced transgenic (PCR-positive) events	Transformation frequency, % ^b^
				Total	PCR-positive for *BAR*		
Type I	1	82	9	10	9	8	9.8
	2	83	6	7	6	5	6.0
	3	50	3	3	3	3	6.0
	4	80	11	12	11	10	12.5
	5	58	11	11	11	11	19.0
	6	100	18	24	23	17	17.0
	7	50	9	9	9	9	18.0
	8	150	24	25	23	22	14.7
**Total / Mean**		**653**	**91**	**101**	**95**	**85**	**12.9**
Type II	1	50	0	0	0	0	0.0
	2	100	2	2	2	2	2.0
	3	48	0	0	0	0	0.0
	4	103	2	2	2	2	1.9
	5	100	9	11	10	8	8.0
	6	75	4	6	6	14	5.3
**Total / Mean**		**476**	**17**	**21**	**20**	**16**	**3.4**

^a^Type I – nodular white morphogenic calli, Type II – nodular translucent morphogenic calli

^b^calculated as percentage of the independent bombarded explants produced at least one PCR- positive transgenic plant T_0_

#### ME-derived tissues

To initiate morphogenic structures from mature embryos of emmer wheat ‘Runo’ we used the endosperm-supported approach [22]. This approach is based on the application of a high Dicamba concentration (10–12 mg/l) to stimulate the formation of morphogenic structures from injured ME connected with a mother seed. The preliminary research showed that the in vitro competence of ME of emmer wheat ‘Runo’ is acceptable, but the size and quality of induced morphogenic structures is the subject for improving [19]. To achieve a better quality of induced ME-derived calli for transformation and to promote the formation of transgenic structures, a plant growth regulator Daminozide was used. One week prior to bombardment 234 morphogenic structures produced by ME were transferred onto medium supplemented with 2 mg/l 2,4-D and 25 mg/l Daminozide. For comparison, the other part of calluses (193 pieces) was cultivated in the medium supplemented solely with 2,4-D.

After being bombarded, ME-derived morphogenic tissues displayed a similar pattern of transient and stable *GFP* gene expression as the IE-derived morphogenic calluses. No evident variation in GFP fluorescence was observed between the calluses cultivated on two media during the two selective subculturings. When ME-derived calli were subcultured on proliferation and differentiation media, a trend for more efficient formation of transgenic structures was observed in the case of Daminozide application. Unlike IE-derived explants, ME-derived calli of emmer wheat did not produce large *GFP* expressing clusters. As a result, only single plantlets were identified in several bombardment experiments after three months of selection (Table 2). Of these, five events were regenerated when Daminozide was added to the medium, and only one transgenic event was recovered without additive. Selected plantlets successfully hardened-off in the greenhouse. PCR screening of the leaf samples from mature plants indicated that all six events contained the *BAR* and the *GFP* genes (Additional file 1: Figure S1). Thus, inclusive of Daminozide resulted in raising transformation frequencies from 0.6 to 2.1% (Table 2).

**Table 2:** Efficiency of transgenic plant production after biolistic- mediated transformation of mature embryo-derived tissues of emmer wheat ‘Runo’ (*T.dicoccum* (Schrank))

Callus selection medium^a^	Experiment No.	No. independent explants bombarded	No. independent explants produced plantlets after dual GFP + PPT selection	No. putative transgenic plants selected in vitro		No. independent explants produced transgenic (PCR-positive) events	Transformation frequency, % ^b^
				Total	PCR-positive		
Daminozide 0 mg/l	1	50	0	0	0	0	0.0
	2	25	0	0	0	0	0.0
	3	48	0	0	0	0	0.0
	4	45	1	1	1	1	2.2
	5	25	0	0	0	0	0.0
**Total / Mean**		**193**	**1**	**1**	**1**	**1**	**0.5**
Daminozide 25 mg/l	1	25	0	0	0	0	0.0
	2	37	0	0	0	0	0.0
	3	25	1	1	1	1	4.0
	4	52	0	0	0	0	0.0
	5	45	2	2	2	2	4.4
	6	50	2	2	2	2	4.0
**Total / Mean**		**234**	**5**	**5**	**5**	**5**	**2.1**

^a^MS basal salts and vitamins, 150 mg/l L-asparagine, 3 mg/l 2,4-D, 3 mg/l PPT

^b^calculated as percentage of the independent bombarded explants produced at least one PCR-positive transgenic plant T_0_

#### Characterization of transgenic events

**Fig. 2:**
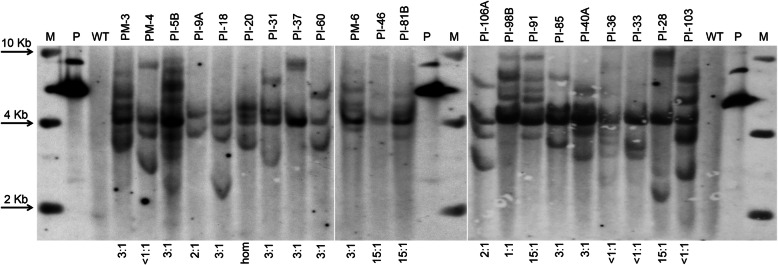
Example of Southern blot analyses of genomic DNAs from PCR- positive T_0_ transgenic plants of emmer wheat ‘Runo’ generated after biolistic-mediated transformation using IE-derived explants (labeled as PI) and ME-derived explants (labeled as PM); genomic as well as plasmid control DNA was digested with *Hind*III and the membrane was probed using the 510-bp *Ubi1*-*BAR* probe; lane P, psGFP-BAR (1.0 ng); lane WT, negative control representing DNA from untransformed ‘Runo’; under the gel, the inheritance of *GFP* expression in T_1_ seedlings of T_0_ primary plant is indicated

Integration of the foreign sequences into the genome of transgenic emmer wheat lines was further confirmed by Southern blot analysis. Unique bands were detected in each transgenic event (Fig. 2; Additional file 2: Figure S2), while the number of hybridising bands significantly varied. For the majority of T_0_ plants, the hybridisation pattern was predominantly complicated, with an average of four to six signals detected. Several lines contained additional hybridising bands of variable molecular weight, indicating the presence of multiple rearranged or truncated copies of introduced cassette. Less than 20% of primary events had one to three copies of transgenes in their genomes.

All the primary transgenic plants were cultivated in a controlled greenhouse to observe morphological changes and to produce seeds for segregation analysis. All 107 independent transgenic events, including IE- and ME-derived plants, developed into phenotypically normal plants that produced from 5 to 20 spikes with a broad range of fertility (Table 3). Nine primary events were completely sterile as no seeds were produced. Several independent plants were partially fertile, producing less than 30 seeds per plant, whereas the majority of the primary transgenic lines (86%) displayed the typical set of seeds (Table 3).

**Table 3:** The seed production in primary transgenic events of emmer wheat ‘Runo’ (*T.dicoccum* (Schrank)) produced after biolistic-mediated transformation of embryo-derived tissues ^a^

Transgenic events quality	Total No.	Percentage
Independent T_0_ events with *GFP* expression	107	
Non-fertile T_o_ events (no seeds were produced)	9	8.4
Transgenic T_0_ events with low fertility (1–30 seeds per plant)	6	5.6
Transgenic T_0_ events with normal fertility (more than 30 seeds per plant)	92	86.0

^a^Event = independent IE or ME, produced transgenic plant(s)

**Fig. 3:**
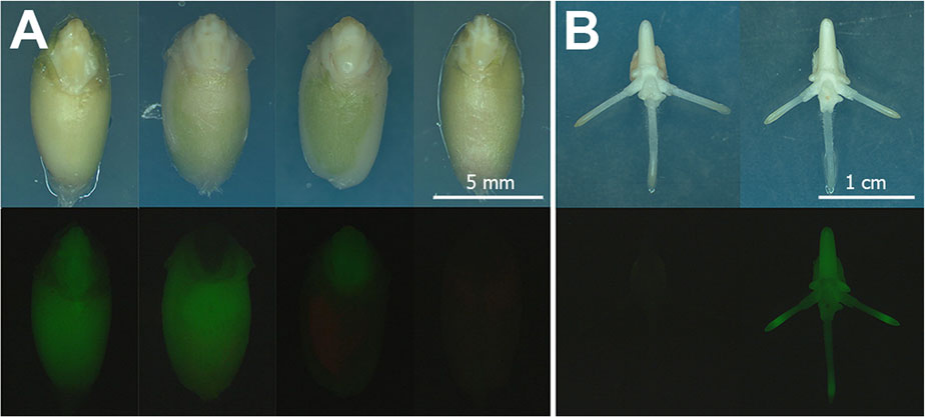
Analysis for the inheritance of GFP fluorescence in T_1_ progeny. **a**. Four variants of GFP fluorescence in T_1_ seeds, left to right: the seed displaying the expression in embryo and endosperm, the seed without *GFP* expression in embryo showing endosperm fluorescence, the seed demonstrating fluorescence in T_1_ embryo, but lacking it in the endosperm, the seed that did not inherit the expression of *GFP* gene. **b**. Inheritance of GFP fluorescence in T_1_ seedlings of a primary transgenic plant, 3 days of in vitro culture of isolated embryos. Tissues were photographed under white light or UV light using the GFP filter set (EX BP 470/40, BS FT 495, EM LP 550)

All primary transgenic plants produced seeds were further evaluated for the inheritance using GFP fluorescence as an indicator of the transgenes segregation. To accelerate the analysis, zygotic T_1_ embryos were isolated from immature seeds (around 20 days post-anthesis) and examined for GFP fluorescence in vitro (Fig. 3). Additionally, *GFP* expression in cells of endosperm was analysed. Four variants of GFP fluorescence were observed. These include the *GFP* expression in both embryo and endosperm, the independent GFP fluorescence in embryo or endosperm, and the lack of transgenic trait inheritance (Fig. 3a,b). Depending on the availability, from 36 to 360 T_1_ seeds harvested from an independent transgenic event were evaluated using chi-square analysis for goodness-of-fit corresponding to three categories of segregation patterns: 1:1, 3:1 and 15:1 (Table 4, Additional file 3: Table S1).

**Table 4:** Summary of segregation analysis of *GFP* expression in T_1_ progeny (embryos) and seeds (endosperms) of T_0_ transgenic events of emmer wheat ‘Runo’ (*T.dicoccum* (Schrank)) ^a^

Visual *GFP* expression segregation ratio (positive:negative)	Endosperms		Embryos		Events with the same segregation in embryos and endosperms	
	No. independent T_0_ events	Percentage	No. independent T_0_ events	Percentage	No. independent T_0_ events	Percentage
No. *GFP* expression	1	1	6	7	–	–
< 1:1	11	12	17	18	5	5
1:1	19	21	16	17	10	11
1:1–3:1	15	16	8	9	4	4
3:1	32	35	32	35	26	28
3:1–15:1	7	8	3	3	1	1
15:1	5	5	8	9	5	5
> 15:1	2	2	2	2	2	2
Total events with the same segregation in embryos and endosperm					53	58
Total No. events analysed	92		92		92	

^a^Only events with normal fertility are presented, progeny were categorized into the respective classes by chi-square analysis (*P* > 0.05)

Most of the seeds displayed fluorescence in both embryo and endosperm. A part of T_1_ seeds displayed *GFP* expression only in endosperm. In five lines all T_1_ embryos were completely silenced despite the expression in endosperms. A limited number of seeds had silenced endosperm, while embryo/seedling had distinctive fluorescence. Due to observed differences, only 53 out of 92 T_0_ plants displayed the equal inheritance of fluorescence in both embryos and seeds (Table 4, Additional file 3: Table S1). Among the analyzed plants, seedlings of 32 primary plants showed segregation for *GFP* expression that statistically fit the Mendelian 3:1 ratio for a single dominant locus (Table 4, Additional file 3: Table S1). According to chi-square analysis, eight T_1_ progenies showed segregation of *GFP* expression as two functional loci. There were 16 lines where a non-Mendelian segregation ratio of 1:1 was observed. In the remainder of lines, the actual segregation patterns for GFP fluorescence did not fit into either of the above three ratios. A significant number of independent transgenic T_0_ lines (18%) displayed a skewed segregation pattern (< 1:1) because most of the T_1_ progeny silenced of *GFP* expression.

There were no clear correlations between numbers of functional loci predicted by the Southern blot hybridization and segregation ratios. Although a 3:1 segregation ratio for the *GFP* gene expression was observed in one third of the events, this result was not consistent with Southern blots data (Fig. 2), since most of the events had a variable number of insertions (from 2 to 7). Similarly, according to the Southern blot DNA evaluations, the primary plants that inherited the expression with a ratio close to 15:1 usually had more than two hybridizing bands (Fig. 2). One of the T_0_ lines, PI-20, was homozygous as all 147 T_1_ seedlings and seeds inherited expression, indicating that probably each of the four detected insertions inherited as an independent functional locus. Despite an inconsistency between the number of insertions detected by Southern hybridization and the actual GFP fluorescence in T_1_ progenies, more than a third of the independent events (40 out of 92) successfully inherited transgenic expression in accordance with a classical Mendelian distribution (3:1 and 15:1), that later enabled the detection of homozygous T_2_ populations with a stable expression (data not shown).

### Generation of transgenic plants of *Triticum timopheevii*

Our previous assays showed that Timopheevi wheat is recalcitrant species with low morphogenic abilities of IE [20]. Based on the positive experience with ME-derived cultures of emmer wheat and our previous experience with IE tissue culture of einkorn wheat [21], we designed several induction media containing various concentrations of Daminozide to examine the morphogenesis and regeneration of transgenic plants from IE embryos of Timopheevi wheat. We found that the presence of Daminozide in callus induction medium had a beneficial effect on the emergence of morphogenic structures in cultured IE (Table 5). Compared to primary induction medium containing only 2,4-D, the percentage of embryogenic/organogenic callus formation on induction media additionally supplemented with 12.5 or 25.0 mg/l Daminozide increased from 30.2 to 57.5% or 65.4%, correspondingly.

**Table 5:** Efficiency of transgenic plant production after biolistic- mediated transformation of immature embryo-derived tissues of Timopheevi wheat (*T.timopheevii* Zhuk)

Callus induction/selection medium^a^	No. IE explant cultured	Morphogenic callus formation (%)^b^	No. bombarded explants	No. explants produced transgenic plants after dual GFP + PPT selection	Transformation efficiency (%)
Daminozide 0 mg/l	845	30.2a	255	0	0.0
Daminozide 12.5 mg/l	652	57.7b	376	2	0.5
Daminozide 25.0 mg/l	538	65.4b	352	0	0.0

^a^MS basal salts and vitamins, 150 mg/l L-asparagine, 3 mg/l 2,4-D; 3 mg/l PPT was additionally added into selection medium

^b^Means having the same letter in the column has no significant differences according to Duncan’s multiple range test (*P* < 0.05)

**Fig. 4:**
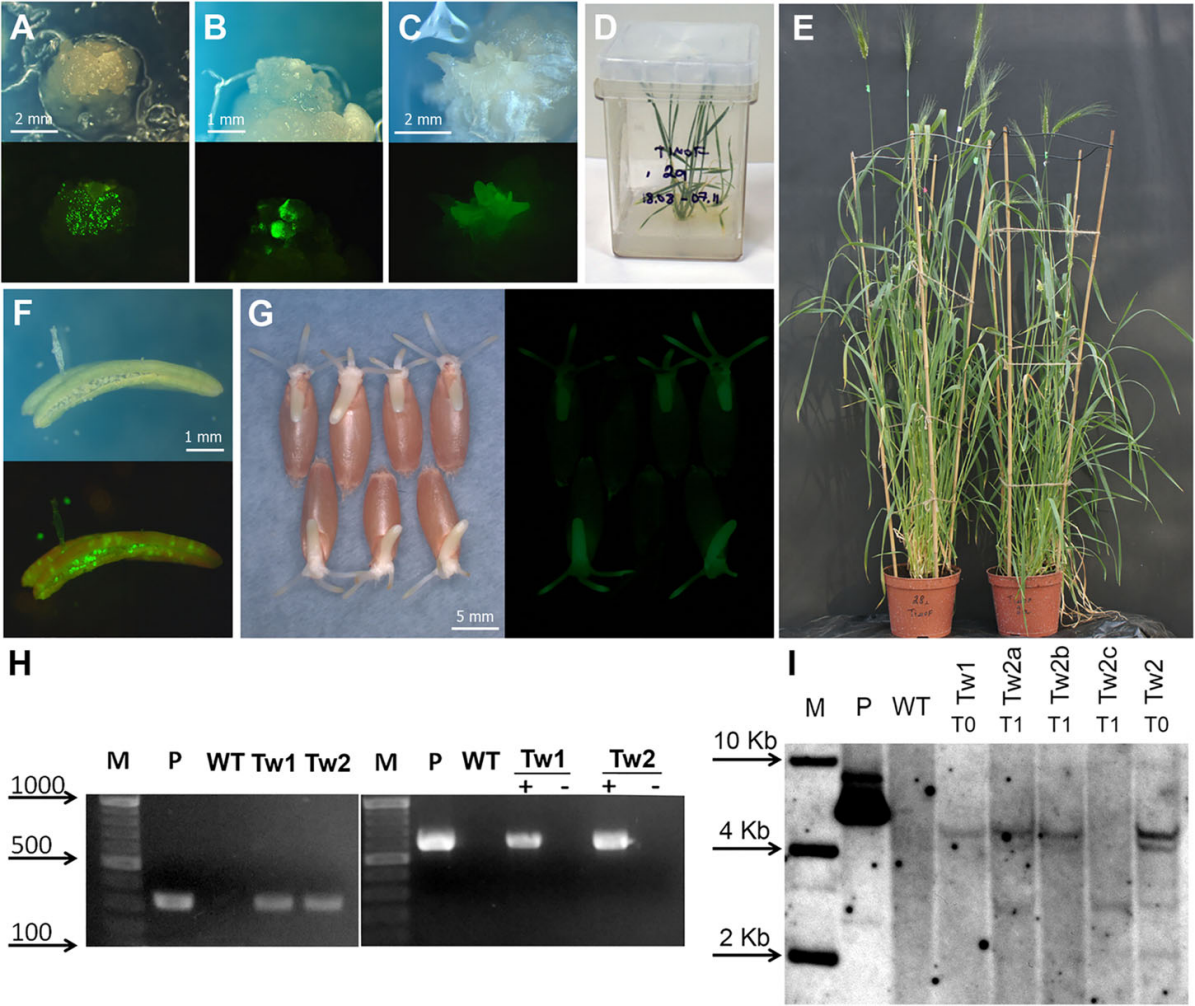
Production and characterization of transgenic plants of *T. timopheevii* (Zhuk.). **a.** Transient *GFP* gene expression in cells of explant subjected to bombardment, 16 h after the plasmid delivery. **b.** Formation of GFP-positive morphogenic callus, 40 days of culture. **c.** Fluorescent embryoids, 65 days of culture. **d.** The putative transgenic plant of *T. timopheevii* before transfer to soil. **e.** Tiller set of primary transgenic plants. **f.** *GFP* expression in anther and pollen grain of primary transgenic wheat plant Tw1. **g.** Inheritance of *GFP* expression in T_1_ seeds of primary transgenic plant Tw2. **h.** Primary plants were analyzed for the presence of *BAR* gene by PCR amplification (left panel) and *GFP* expression by RT-PCR (right panel); lane M, DNA ladder as molecular weight marker; lane P, psGFP-BAR; lane WT, untransformed *T. timopheevii* plant; lanes Tw1 and Tw2 represent putative transgenic plants; +, a sample with addition of reverse transcriptase; −, the same sample without addition of reverse transcriptase. **i**. Southern blot analyses of genomic DNAs from PCR-positive primary transgenic plants Tw1 and Tw2 (T0) and three progeny plants (T1) of the transgenic line Tw2; genomic as well as plasmid control DNA was digested with *Hind*III and the membrane was probed using the 510-bp *Ubi1*-*BAR* probe; lane P, psGFP-BAR (1.0 ng); lane WT, negative control representing DNA from untransformed *T. timopheevii* plant. Tissues (**a,b,c,f,g**) were photographed under white light or UV light using the GFP filter set (EX BP 470/40, BS FT 495, EM LP 550)

The produced morphogenic calluses displayed an acceptable level of transient *GFP* expression next day after biolistic-mediated delivery of psGFP-BAR plasmid (Fig. 4a). After 30–60 days of the selection, most of the bombarded explants lost *GFP* expressing cells, and only a limited number of foci with a bright fluorescence was found (Fig. 4b). As a result of the five independent bombardment experiments involving 50–130 explant for each variant of induction medium, only two independent GFP- positive organogenic structures were survived to the end of selection on the differentiation medium (Fig. 4c). Both fluorescent structures were developed from IE-derived callus induced on the medium supplemented with 12.5 mg/l Daminozide. They successfully regenerated plants, which later readily proliferated and rooted in vitro (Fig. 4d).

Two primary transgenic plantlets of Timofeevi wheat, designated as Tw1 and Tw2, easily hardened-off in the greenhouse and grew to maturity producing spikes (Fig. 4e). Both plants were positive for the *BAR* insertion according to PCR and Southern blot (Fig. 4i,h; Additional file 2: Figure S2), resulting in an average transformation efficiency of 0.5% (Table 5). Hybridization analysis of genomic DNA samples from Tw1 and Tw2 transgenic plants yielded different hybridization patterns confirming their independence. One of the plants, Tw1, had a single insertion, while the second primary plants displayed at least four independent site insertions (Fig. 4i). Screening for *GFP* gene expression using cDNA prepared from mRNA leaf extracts of Tw1 and Tw2 also showed the amplification, while the mRNA sample of wild-type plant did not produce the expected fragment (Fig. 4h; Additional file 2: Figure S2). In both transgenic events, the expression of *GFP* was found in various tissues of mature plants, including anthers and pollen (Fig. 4f).

Despite the formation of a sufficient number of spikes, the primary plant Tw1 was sterile and did not set seeds; in contrast, plant Tw2 produced several hundred seeds. T_1_ seedling germinated from Tw2 seeds, inherited expression of introduced transgenes by exhibiting a bright GFP fluorescence, clearly distinguishable from the negative T_1_ counterparts (Fig. 4g). Analysis for transgene segregation showed that from 232 seedlings subjected to GFP analysis, the expression was found in 213 seedlings, giving the ratio 11:1 (positive:negative). According to Chi-square test, the observed ratio is in agreement with the Mendelian 15:1 segregation ratio (χ2 = 1.49 < χ2_tabl_ = 3.84, at *P >* 0.05). The inheritance data were correlated with the Southern blot analysis of transgenic T_1_ seedlings for *BAR* segregation. As shown in Fig. 4i (Additional file 2: Figure S2), three different patterns of hybridization among segregating progeny were observed: one was similar to the parent plant (lane Tw2a), while two others represent independent segregation of hybridization signal(s) (lanes Tw2b and Tw2c). This result, together with GFP segregation data, indicates the presence of two independent functional transgene loci in transgenic line Tw2.

## Discussion

After the first reports describing the production of transgenic wheat plants, the transformation methodology has taken a significant step in improving the efficiency of transgenic event production using a biolistic-mediated delivery [11]. Nevertheless, wheat is regarded as a recalcitrant crop for genetic transformation and new approaches should focus on increasing the number of cultivars available for the successful delivery of various heterologous sequences. This is especially true for tetraploid wheat germplasms because still a limited number of varieties were successfully transformed [11]. Since the production of transgenic plants requires a proper adjustment between the regeneration and transformation methods, various factors have been taken into consideration over the past decade to improve transformation efficiency for a range of cultivars including the ability of the genotype to regenerate plants, the explant type, the bombardment conditions, and the selection strategy.

In the present study, attention has been paid to the quality of the morphogenic callus used as the starting materials for establishing an effective method for the production of transgenic plants of tetraploid wheat species. Experiments to compare two types of IE-derived nodular callus of emmer wheat showed a significant increase in the number of stable transgenic events when hard white compact nodular callus was bombarded (Type I). The proper choice of tissue for bombardment coupled with a dual selection strategy (*GFP* + *BAR*) allowed generation independent events of emmer wheat ‘Runo’ with an average frequency of 12.9%. Such efficiency is one of the highest efficiencies achieved for tetraploid germplasms of wheat. For comparison, the efficiency of gene gun-mediated transformation for ‘Svevo’, the most popular tetraploid wheat cultivar for genetic engineering, is fluctuated in the range of 0.3 to 3.5% [11]. A relatively high transformation rate of 4.6% was recently reported for Egyptian durum wheat ‘Bani Suef 6’ [23], while efficiency across the other tetraploid wheat genotypes was 0.5–2.8% for 'Varano' and 'Mexicali', 0.8–1.3 for D5c31YN S48, 2.1–3.4% for PDW215, 1.6% for MG4343A, and less than 1% for 'Ofanto', 'Creso' and 'Luna' [11, 14, 24–27]. A fairly high transformation frequency of 4.3–4.9% was reported for Indian emmer wheat DDK1001; although considering the problems with transferring of regenerated in vitro plant to the greenhouse (only 13 out of 19 plantlets survived) and low plant fertility (8 out of 13 plants were sterile), the real efficiency was much lower as claimed [12, 13]. Although a significantly higher number of hexaploid bread wheat cultivars were subjected to biolistic-mediated DNA delivery, transformation rates higher than 10% were also rarely reported [11]. In the case of the dominated model variety ‘Bobwhite’ it was possible to achieve transformation efficiency up to 60% of in particular experiments [28], however, in the most of the studies it was ranged between 0.5 and 10% [11].

The fact that the choice of a suitable callus type is one of the critical points for improving the production of transgenic cereals in embryo-derived culture for a given genotype was already documented [29–31]. Here we relied on in the natural ability of the cultivated IE embryos of ‘Runo’ to produce morphogenic structures of different quality. In the case of Timopheevi wheat, modification of the induction medium was required to improve the quality of the bombarded calli. In general, *T. timopheevii* demonstrates low abilities to produce regenerable nodular callus; moreover, among regenerated plants, a significant number of albino-plants are formed [19, 20]. Previously we have found that the including of Daminozide in combination with various auxin-like substances was highly beneficial to stimulate the formation of morphogenic calli in recalcitrant diploid and hexaploid wheat [21, 32], mostly by increasing the portion of hard nodular structures. Daminozide was acted in wheat tissue culture similarly to various plant growth retardants and ethylene inhibitors, which were previously reported to promote somatic embryogenesis and plant regeneration in barley [33], maize [34] and loblolly pine [35]. At the same time, a higher level of Daminozide converted white morphogenic callus into dormant non-regenerative clumps [21, 32], so a concentration adjustment is needed to achieve an acceptable level of plants regeneration. In the present study, the addition of low concentrations of Daminozide (12.5–25 mg/L) increased the amount of morphogenic callus in cultured IE of *T. timopheevii* by at least two times. Daminozide supplementation further resulted in the generation of independent transgenic plants of this cultivated tetraploid species. Even though the transformation efficiency was only 0.5% using one of induction media variants, this is the first successful report for generating transgenic plants of the wheat species with the G-genome.

The application of Daminozide was also beneficial for improvement transformation rate when ME-derived tissues of emmer wheat ‘Runo’ were used as explants for plasmid delivery. IE-mediated genetic transformation of wheat, including the method described here for emmer wheat, usually requires 7–8 months to establish T_0_ plants in the greenhouse. Approximately half of this time is growing donor plants for isolation of IE, while the development stage of IE to achieve the highest morphogenic response is restricted to one-two days. Since mature seeds are available around the year without restriction, an efficient and reproducible method for generating stable transgenic events from tissues of mature seeds has an enormous potential to serve as a simple and easy way to use in various experimental purposes. Several attempts were carried out by various research groups to produce transgenic wheat plant using mature seeds as starting explants, mostly by application of *Agrobacterium*-mediated transformation approach [11]. In our previous experiments, we tried to apply the endosperm-supported approach for the induction of morphogenic structures in hexaploid bread wheat for further use in biolistic-mediated transformation [33]. The resulted overall efficiency of transgenic event production, however, was insufficient to be used routinely for various experimental purposes. In the present study, when a similar protocol was used for emmer wheat ‘Runo’, an equal transformation rate of 0.5% was obtained. To get high transformation frequency, we applied low Daminozide concentration to stimulate the conversion of morphogenic calli induced from mature seeds into a more perceptive tissue for a gene-gun delivery of plasmid DNA. Supplementation of this growth regulator into the pre-culture and the callus selection media provided a noticeable increase in the yield of independent transgenic events. The resulting transformation efficiency, with an average of 2.5%, was comparable or even higher with previously reported results for bread wheat cultivars when ME or ME-derived tissues were used for biolistic-mediated transformation [11, 36].

Our findings support the trend towards multiple integrations of foreign sequences after the biolistic-mediated DNA delivery, previously reported in transgenic *Triceae* species, such as einkorn wheat [37], barley [38], durum wheat [26, 39], bread wheat [28, 34, 40–42] and triticale [43]. In the present study, the majority of the produced transgenic emmer wheat plants had from 3 to 8 inserts. The complexity of integration pattern, however, was not directly correlated with the actual segregation of transgene expression. The significant part of independent events with varying patterns of multiple insertions (30.8%) inherited the detectable expression of *GFP* as a single functional locus (Fig. 2). Nonetheless, the considerable number of independent events displayed deviations from Mendelian segregation in the T_1_ progeny (Table 3). The observed variations, however, were similar to those observed previously in transgenic wheat [40, 42, 44].

A noticeable part of independent events (44%) demonstrated the different patterns of transgenic trait segregation in embryos and endosperms of T_1_ seeds. For example, the progeny of six primary plants (7%) lacked *GFP* expression in T_1_ seedlings, while exhibiting it in the endosperm. In general, the GFP fluorescence in the endosperm was found in 8892 seeds, while only 7915 T_1_ embryos/seedlings were positive for *GFP* expression. Such inconsistency was not previously described in deep for other cereals since the expression of transgenes was generally directed to vegetative tissues and mature plants. Since the endosperm forms during double fertilization, its triploid nature might create unusual gene dosage and gene interactions, especially in polyploid species like wheat [45]. Taking into account that the biolistic-mediated transformation may induce large severe rearrangements in cereal genomes [46], the uneven contribution of maternal:paternal genomes may affect the dosage/combination of transgenic functional/non-functional copies in cells of embryo and endosperm. The correct explanation for the abnormalities of transgene inheritance remains elusive, and various factors, including the characteristics of the introducing vector, position effects, and genetic background, are supposed to influence the actual transmission to the progeny [47]. There is general agreement that the transgene segregation could be more predictable in the case of a single insert or a low number of insertions. We believe that some methodological approaches, such as the use of dephosphorylated gene cassette [41] or linear form of the construct [48], and the PEG/Mg2+ coating procedure [49] will be useful for combination with the method presented here to increase the number of transgenic events with simple integration patterns.

## Conclusion

In the present study, an effective biolistic-mediated method for the production of numerous independent transgenic events of tetraploid emmer wheat ‘Runo’ is described. We found that white nodular embryogenic/organogenic calli produce more transgenic events than softer and watered morphogenic structures. This phenomenon could be considered in experiments directed to discover new wheat cultivars suitable for large-scale transformation studies using gene gun DNA delivery. The hormonal modification of culture medium, described in the present study, could be taking into account to improve the quality of the induced morphogenic tissue of wheat as it ultimately facilitates the transformation process. Due to this, transgenic plants of *T.timopheevii*, a wheat species with G genome, were obtained for the first time. We also describe an alternative procedure for the production of wheat plants with the use of ME-derived cultures. Given the practical utility of presented procedures, especially in the light of high transformation ability of emmer wheat ‘Runo’, it would be useful to exploit our current findings for future genetic transformation and genome editing studies where large numbers of independent events are required.

## Methods

### Plant material and general conditions

A spring emmer wheat ‘Runo’ (*T. dicoccum* (Schrank), derivative from accession number K-17560), kindly donated by Ludmila A. Bespalova (National Center of Grain named after P.P. Lukyanenko, Krasnodar, Russia), and Timophefeevi wheat (*T. timopheevii* (Zhuk.), accession number К-47793), provided by Prof. Gennady I. Karlov (All-Russia Research Institute of Agricultural Biotechnology) were used in the present study. Donor and transgenic wheat plants were grown in glasshouse conditions under a 16-h photoperiod at 25 ± 2 °C during the day and 20 ± 2 °C during the night. When plants reached the three-leaf stage, they were fertilized every week. Seeds were sown at two weekly intervals to provide a constant supply of immature caryopses for IE isolation.

The basic nutrient medium for experiments consisted of mineral salts and vitamins according to Murashige and Skoog (MS) [50], 30 g L^− 1^ sucrose, 150 mg L^− 1^ asparagine and 7 g L^− 1^ agarose. The pH of the medium was adjusted to 5.8 before autoclaving (120 °C for 20 min). Plant growth regulator solutions were filter sterilized with the use of Millipore filters (0.22 μm) and were added to the autoclaved medium when the temperature was around 40–55 °C. in vitro cultures were kept in the dark at 25 ± 2 °C in Petri dishes and subcultured to fresh medium every three weeks unless otherwise specified.

### Production of transgenic plants of emmer wheat ‘Runo’

**Fig. 5:**
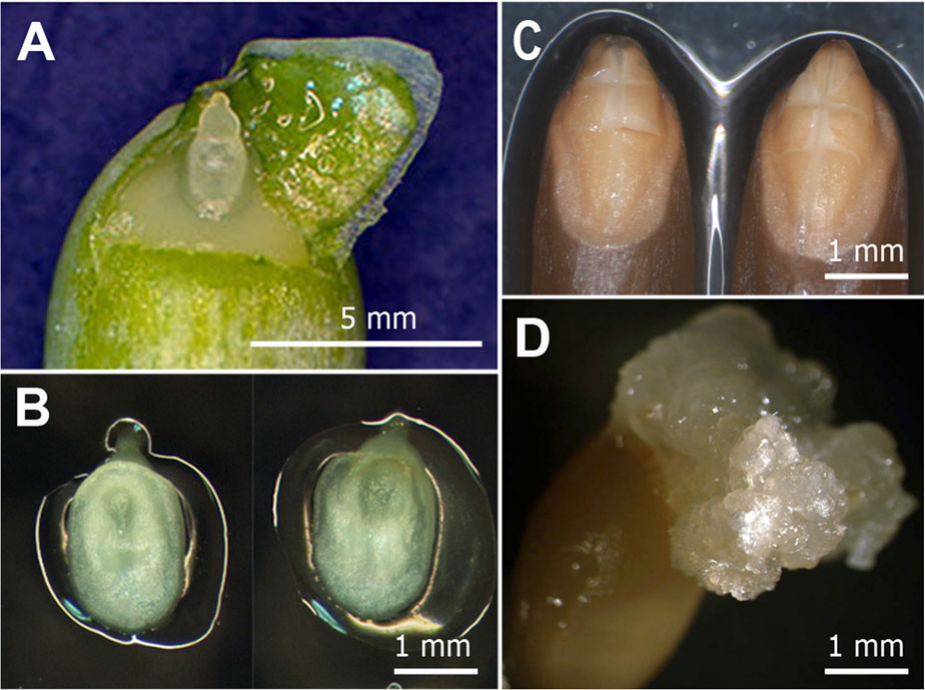
**a**. Isolation of IE of emmer wheat ‘Runo’ from immature caryopse. **b**. Isolated IEs placed scutellum-side up on the induction medium. **c**. Imbibed mature seeds with cutting on ME. **d**. Morphogenic callus from ME (21 days of culture) before the removal and the transfer onto the pre-culture medium

Both IE- and ME-derived morphogenic calluses of emmer wheat ‘Runo’ were used for biolistic-mediated transformation. Immature seeds of emmer wheat were harvested from spikes within 12–14 days after anthesis. Isolated caryopses were soaked firstly in 70% ethanol (v/v) for 2 min and then in the solution containing 16.5% (v/v) commercial bleach (ACE laundry bleach) supplemented with a few drops of surfactant Tween 20 while shaking at 180 rpm for 18 min. Surface-sterilized caryopses were rinsed five times with sterilized water. Translucent immature embryos of 0.75–1.5 mm were dissected from caryopses under a stereomicroscope in laminar flow hood (Fig. 5a) and immediately placed scutellum-side up onto the induction media (Fig. 5b). Culture induction medium (CIM) for immature embryos consisted of basal medium supplemented with 2 mg L^− 1^ 2,4-D. Two target tissues, white (Type I) or translucent (Type II) nodular morphogenic structures raised from immature embryos during 10–15 days of culture (Fig. 1b,c) were used as explants for transformation. After the plasmid delivery, the bombarded explants were moved into callus selection medium CSM, representing CIM supplemented with 3 mg/l PPT. After a new subculture on the fresh CSM, the tissue with GFP fluorescence was separated from the non-morphogenic tissue and sub-cultivated on the proliferation medium (PM) consisted of the basic nutrient medium supplemented with 1 mg L^− 1^ 2,4-D and 3 mg/l PPT. The surviving morphogenic tissues with *GFP* expression were placed into the differentiation medium (DM) to induce the formation of shoot primordial. DM composition was the same as PM, with the exception that the concentration of 2,4-D was reduced to the 0.5 mg/l. Following the 2–3 weeks on DM, GFP- positive embryos/buds were transferred onto a regeneration medium (RM, phytohormone-free basal medium containing 3 mg/l PPT). Explants regenerating putative transgenic plants were subcultured in RM for 10- to 15-day intervals until the plantlets reached approximately 3–4 cm in length. Finally, the developing shoots with roots displaying the GFP fluorescence were individually moved onto Magenta boxes containing 100 mL of phytohormone-free basic medium supplemented with 2 mg/l PPT and cultured under the light (16 h photoperiod, 100 μmol m^− 2^ s^− 1^) at 24 ± 2 °C. Rooted plants with a height of at least 10 cm were transplanted to the soil as described [20].

ME-derived culture of emmer wheat was initiated from dry seeds obtained from plants grown in the previous crop season in a glasshouse. Mature dry seeds were surface-sterilized by soaking in 50% (v/v) commercial bleach (ACE laundry bleach) for 30 min at 150 rpm, rinsed several times in sterile water and imbibed in sterile water for 3 h at room temperature with 3–4 water changes to remove residual bleach from seeds. To initiate callus, ME were aseptically cross-cut with a scalpel in such way, that the connection between the shoot and radical would be damaged, while the embryos were not detached from the seeds (Fig. 5c). The whole seeds with injured embryos were transferred into the MS medium containing 20 g L^− 1^ sucrose, 7 g L^− 1^ agarose, 12 mg/l 3,6-dichloro-o-anisic acid (Dicamba) and 0.5 mg/l indoleacetic acid (IAA). After callusing for about 2–4 weeks, developed nodular compact structures (Fig. 5d) were removed from cultured seeds and pre-incubated before bombardment for 5–7 days on the CIM or SIMD media. SIMD is CIM additionally supplemented with 25 mg/l succinic mono*-*N*,*N*-*dimethylhydrazide (Daminozide). After the plasmid delivery, bombarded explants were correspondently transferred to the callus selection media CSM (CIM + 3 mg/l PPT) or CSMD (CIM + 25 mg/l Daminozide + 3 mg/l PPT). Daminozide was removed after one subculture, and all explants were transferred onto the fresh CSM. A further selection of transgenic tissue and plants was carried out the same way as described above for IE-derived tissues of emmer wheat using the same media (PM, DM and RM).

### Production of transgenic plants of Timopheevi wheat

The sterilisation and isolation of IE of *T.* *timopheevii* (Zhuk.) were carried out the same way as described above for emmer wheat IE with the exception that immature seeds were harvested from spikes 9–10 days post-anthesis. Isolated IE were cultured on three induction media (IM), representing the basal nutrient medium (MS mineral salts and vitamins, 30 mg/l sucrose, 150 mg/l asparagine) supplemented with 3 mg/l 2,4-D and various concentrations of Daminozide: IM0, IM12 or IM25, containing, correspondently, 0, 12.5 or 25 mg/l Daminozide. As morphogenic calluses formed, they were used for transformation. After the plasmid delivery, the bombarded explants were transferred to the same variant of induction medium but additionally supplemented with 3 mg/l PPT to select transgenic tissue. After one subculture all explants were transferred onto a fresh IM0 supplemented with the same level of PPT. A further selection of transgenic tissue and plants was carried out the same way as described above for IE-derived tissues of emmer using PM, DM and RM media compositions.

### Plasmid preparation and biolistic-mediated transformation

Transformation experiments were conducted with a vector psGFP-BAR [51], kindly provided by B.V. Conger, University of Tennessee. The plasmid (Additional file 1: Figure S1) contains the gene of the green fluorescent protein (*GFP*) driven by the rice actin1 (*Act1*) promoter, and the *BAR* gene encoding phosphinothricin acetyltransferase under the maize ubiquitin (*Ubi1*) promoter to confer resistance to the herbicide phosphinothricin (PPT). The plasmid DNA was prepared using a GeneElute HP Plasmid Midiprep Kit (Sigma-Aldrich, USA). For the bombardment, the concentration of the plasmid DNA was adjusted to 1 μg/μl.

A particle inflow gun (PIG) [52] was used to deliver psGFP-BAR plasmid into the wheat tissues. The preparation of tungsten particles and the procedure of bombardment were carried out as described [37]. In all transformation experiments, embryo-derived morphogenic explants were cultured 4–6 h prior to bombardment on the appropriate induction medium (CIM for emmer wheat and IM0 for Timopheevi wheat) supplemented with 0.4 M Mannitol as osmoticum. Explants were located in 1.8-cm-diameter circle at the centre of dishes to avoid the excessive damage of tissues. Each dish, regarded as one replicate, contained 18–26 explants. A helium pressure of 80 Psi and 0.7 μm tungsten particles [37] were used to deliver the plasmid into the wheat cells. After the plasmid delivery explants were maintained on the osmoticum medium for additional 16–20 h and then transferred to the appropriate selection medium containing PPT.

### Analysis of putative transgenic plants

GFP monitoring was performed using a ZEISS SteREO Discovery.V12 microscope equipped with a PentaFluar S 120 vertical illuminator as described [37].

The transgenes integration into the genome of tetraploid wheat plants was examined by PCR and Southern hybridization. Genomic DNA was prepared from leaf tissue using CTAB method. Putative transgenic plants were screened by PCR using gene-specific primers for *BAR* gene (GenBank no. JQ293091; primer pair: 5′-TGC ACC ATC GTC AAC CAC TA-3′, 5′-ACA GCG ACC ACG CTC TTG AA-3′; a 311-bp fragment), and for *GFP* gene (GenBank no. EF090408.1; primer pair: 5′-GCG ACG TAA ACG GCC ACA AG -3′, 5′-CCA GCA GGA CCA TGT GTG ATC G  -3′; a 606-bp fragment). PCR assay was carried out as described previously [53]. Total RNA was extracted using the Aurum Total RNA Fatty and Fibrous Tissue kit (Bio-Rad, USA). The plant RNA was subjected to reverse transcription and the synthesized cDNAs were used as the template for RT-PCR using the same conditions as for the PCR. For DNA hybridization analysis, approximately 30 μg of genomic DNA per sample was digested with *Hind*III. The digested DNA was separated by 0.9% agarose-gel electrophoresis, blotted onto N+ Hybond nylon membranes and hybridized with an alkaline phosphatase probe. The 510- bp probe was generated by PCR from psGFP-BAR plasmid as a template using the primers specific for amplification of sequence consisted of the *Ubi1* promoter fragment (5′-GGA TGA TGG CAT ATG CAG CAG CTA TAT G-3′) and *BAR* gene (5′-CCA GCA GGA CCA TGT GTG ATC G  -3′). Southern blotting was carried out as described [25].

Analysis of transgene segregation was conducted on T_1_ progeny (embryos/seedlings and seeds) via GFP fluorescence. The procedure for the analysis was described previously [37]. Briefly, embryos were isolated from surface-sterilized Т_1_ seeds (20–25 days post-anthesis) and placed on germination medium for monitoring *GFP* expression in various tissues. The Chi-squared (χ2) test was used to interpret the goodness-of-fit of *GFP* segregation between observed and expected distributions against genetic ratios of 1:1, 3:1 and 15:1. Values of χ2 greater than 3.84 indicates that the observed ratio of segregation is different from the expected ratio (at *P >* 0.05).

## Supplementary information

**Additional file 1: Figure S1.** Schematic diagrams of psGFP-BAR vector used for transformation. Red bar represents the position of the probe used for Southern blot analysis. **b,c**. Putative transgenic plants of emmer wheat ‘Runo’ produced within experiments were analyzed for the presence of *BAR* gene by PCR amplification (**b**) and *GFP* expression by RT-PCR (**c**); an example of analysis for a part of plants generated using IE-derived explants (labeled as PI) and ME-derived explants (labeled as PM); lane M, DNA ladder as molecular weight marker; lane P, psGFP-BAR; lane WT, untransformed emmer wheat plant; +, a sample with addition of reverse transcriptase; −, the same sample without addition of reverse transcriptase.

**Additional file 2: Figure S2**. Molecular analysis of transgenic events of emmer wheat and Timopheevi wheat presented in Fig.2 and Fig.4h,i. The raw gels data.

**Additional file 3: Table S1.** Segregation analysis of *GFP* expression in T_1_ progeny of transgenic plants of emmer wheat ‘Runo’.

**Additional file 4: Figure S3.** Molecular analysis of transgenic events of emmer wheat presented in **Figure S1.** The raw gels data.

## Data Availability

The data generated or analyzed during this study are included in this published article and its supplementary files. The datasets analyzed during the current study are available from the corresponding author on reasonable request.

## Abbreviations

*BAR*: Bialaphos resistance gene
GFP: Green fluorescent protein
PIG: Particle inflow gun
PPT: Phosphinothricin
Psi: Pound per square inch
2,4-D: 2,4-dichlorophenoxyacetic acid
ME: Mature embryo
IE: Immature embryo
