# Supplementary material for: A competence of embryo-derived tissues of tetraploid cultivated wheat species Triticum dicoccum and Triticum timopheevii for efficient and stable transgenesis mediated by particle inflow gun
Source: BMC Plant Biol. 2020 Oct 14;20(Suppl 1):442. doi: 10.1186/s12870-020-02580-4 (PMC7557024; doi:10.1186/s12870-020-02580-4)
Supplement: Supplementary file 1 — Additional file 1: Figure S1. Schematic diagrams of psGFP-BAR vector used for transformation. Red bar represents the position of the probe used for Southern blot analysis. b,c. Putative transgenic plants of emmer wheat ‘Runo’ produced within experiments were analyzed for the presence of BAR gene by PCR amplification (b) and GFP expression by RT-PCR (c); an example of analysis for a part of plants generated using IE-derived explants (labeled as PI) and ME-derived explants (labeled as PM); lane M, DNA ladder as molecular weight marker; lane P, psGFP-BAR; lane WT, untransformed emmer wheat plant; +, a sample with addition of reverse transcriptase; −, the same sample without addition of reverse transcriptase. [file 12870_2020_2580_MOESM1_ESM.pdf]

Supplementary Material 1

A competence of embryo-derived tissues of tetraploid cultivated wheat species *Triticum dicoccum* and *Triticum timopheevii* for efficient and stable transgenesis mediated by particle inflow gun

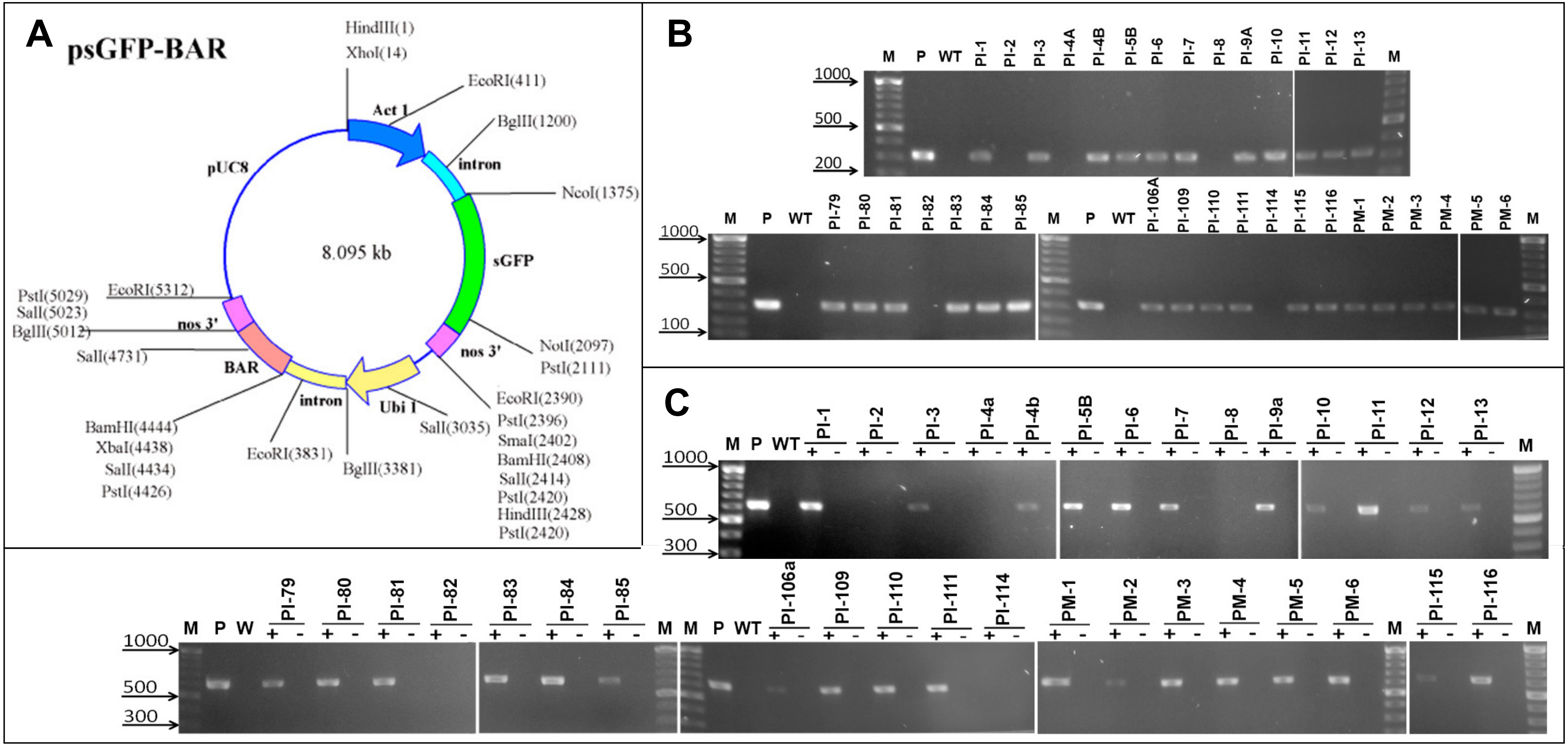

Figure S1. a. Schematic diagrams of psGFP-BAR vector used for transformation. Red bar represents the position of the probe used for Southern blot analysis. **b,c**. Putative transgenic plants of emmer wheat 'Runo' produced within experiments were analyzed for the presence of *bar* gene by PCR amplification (**b**) and GFP expression by RT-PCR (**c**); an example of analysis for a part of plants generated using IE-derived explants (labeled as PI) and ME-derived explants (labeled as PM); lane M, DNA ladder as molecular weight marker; lane P, psGFP-BAR; lane WT, untransformed emmer wheat plant; +, a sample with addition of reverse transcriptase; -, the same sample without addition of reverse transcriptase.
