# Supplementary material for: A competence of embryo-derived tissues of tetraploid cultivated wheat species Triticum dicoccum and Triticum timopheevii for efficient and stable transgenesis mediated by particle inflow gun
Source: BMC Plant Biol. 2020 Oct 14;20(Suppl 1):442. doi: 10.1186/s12870-020-02580-4 (PMC7557024; doi:10.1186/s12870-020-02580-4)
Supplement: Supplementary file 2 — Additional file 2: Figure S2. Molecular analysis of transgenic events of emmer wheat and Timopheevi wheat presented in Fig.2 and Fig.4h,i. The raw gels data. [file 12870_2020_2580_MOESM2_ESM.pptx]

## Slide 1
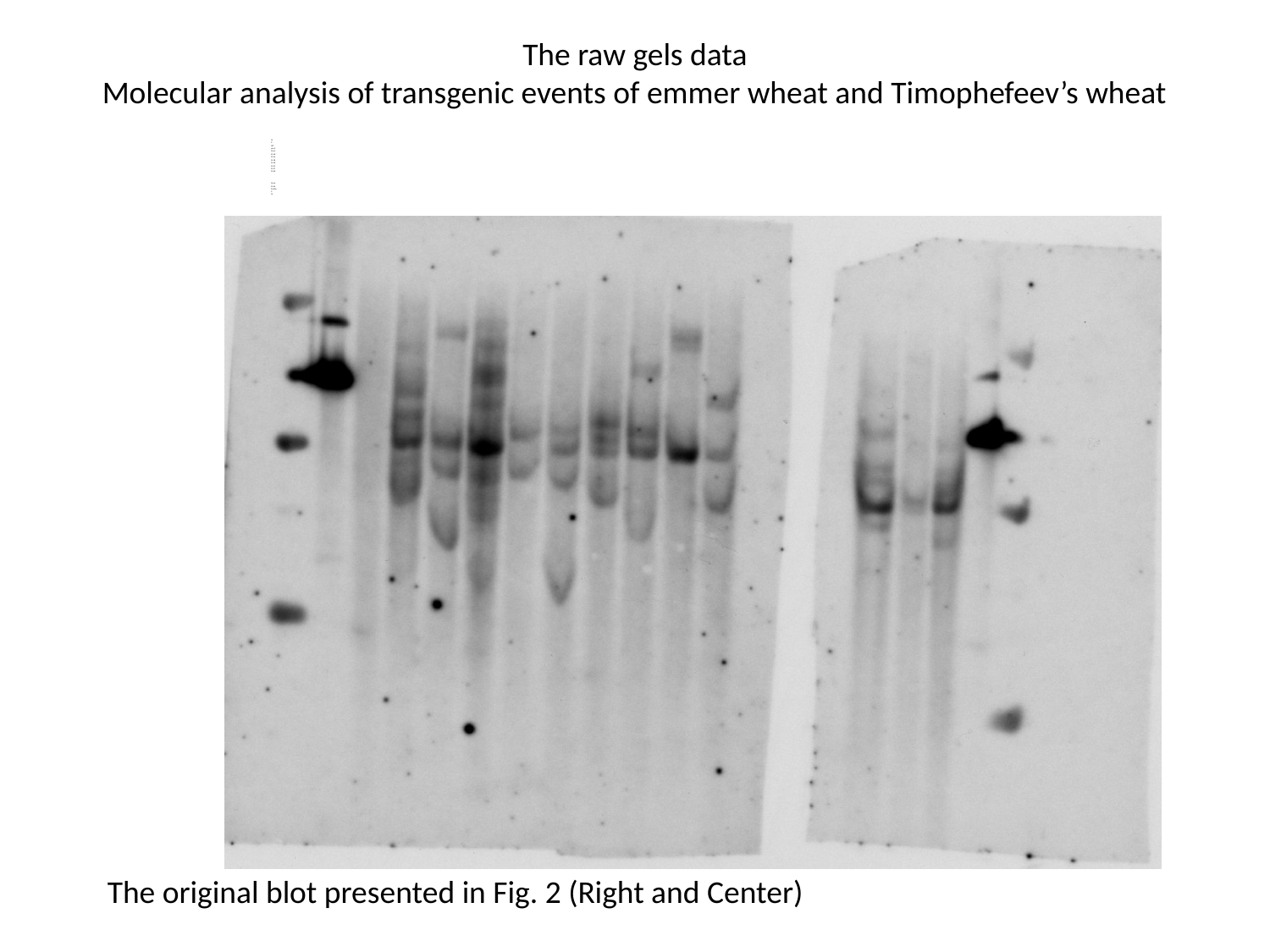

# The raw gels data Molecular analysis of transgenic events of emmer wheat and Timophefeev’s wheat
M
P
WT
pm-3
Pm-4
Pi-5b
Pi-9a
Pi-18
Pi-20
Pi-31
Pi-37
Pi-60
Pm-6
Pi-46
Pi-81b
P
M
The original blot presented in Fig. 2 (Right and Center)

## Slide 2
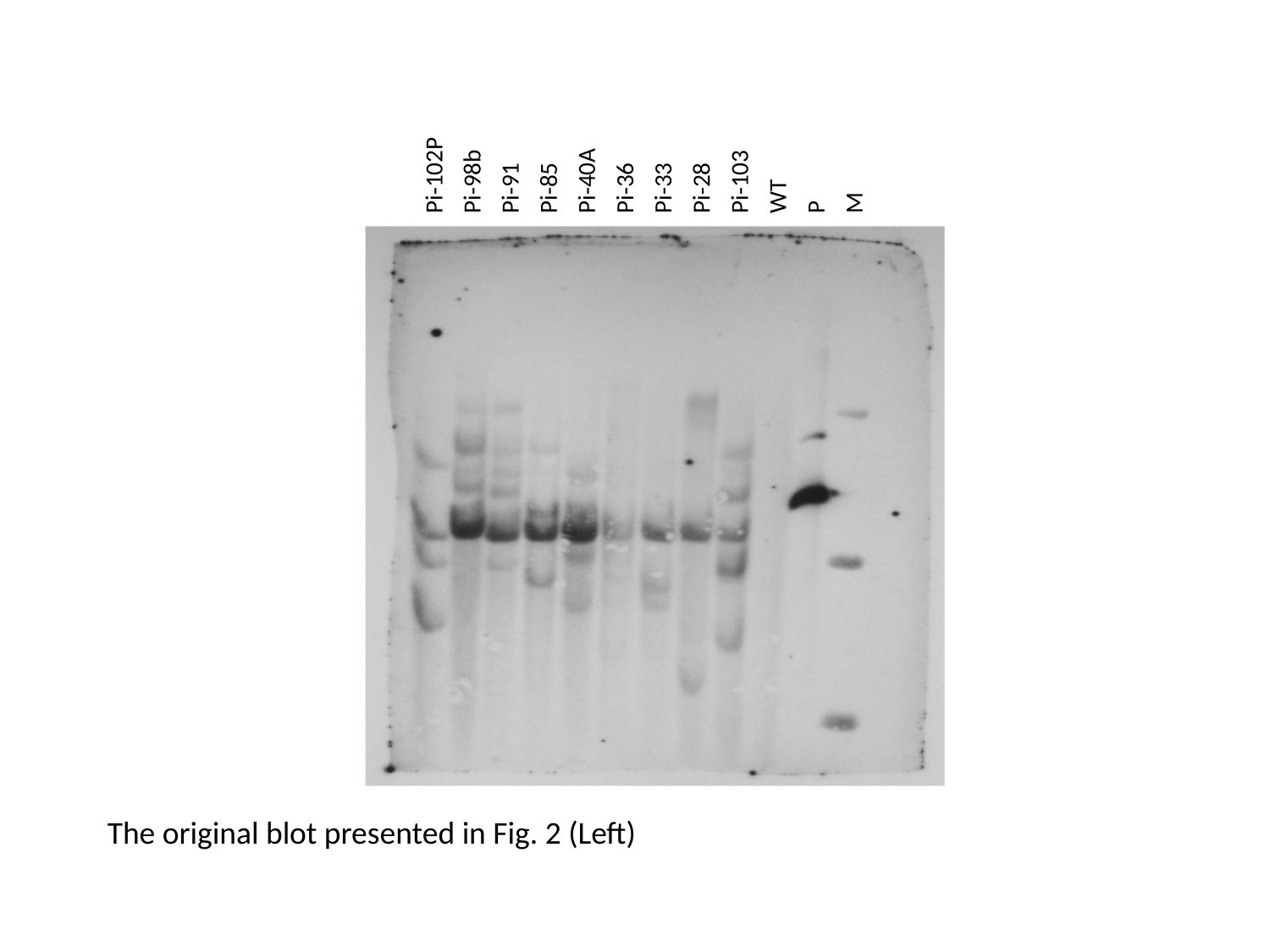

Pi-102P
Pi-98b
Pi-91
Pi-85
Pi-40A
Pi-36
Pi-33
Pi-28
Pi-103
WT
P
M
The original blot presented in Fig. 2 (Left)

## Slide 3
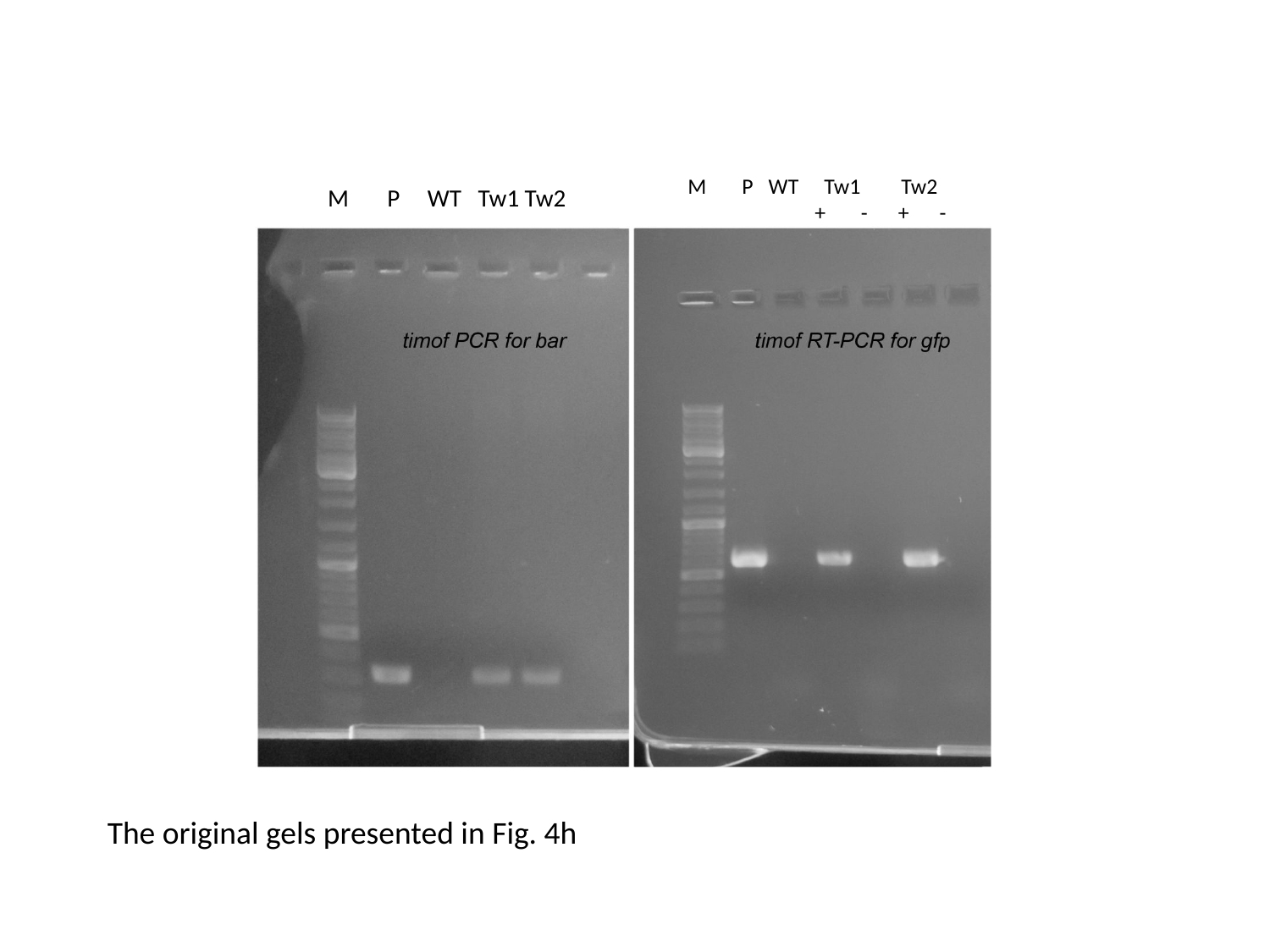

M P WT Tw1 Tw2
 + - + -
M P WT Tw1 Tw2
The original gels presented in Fig. 4h

## Slide 4
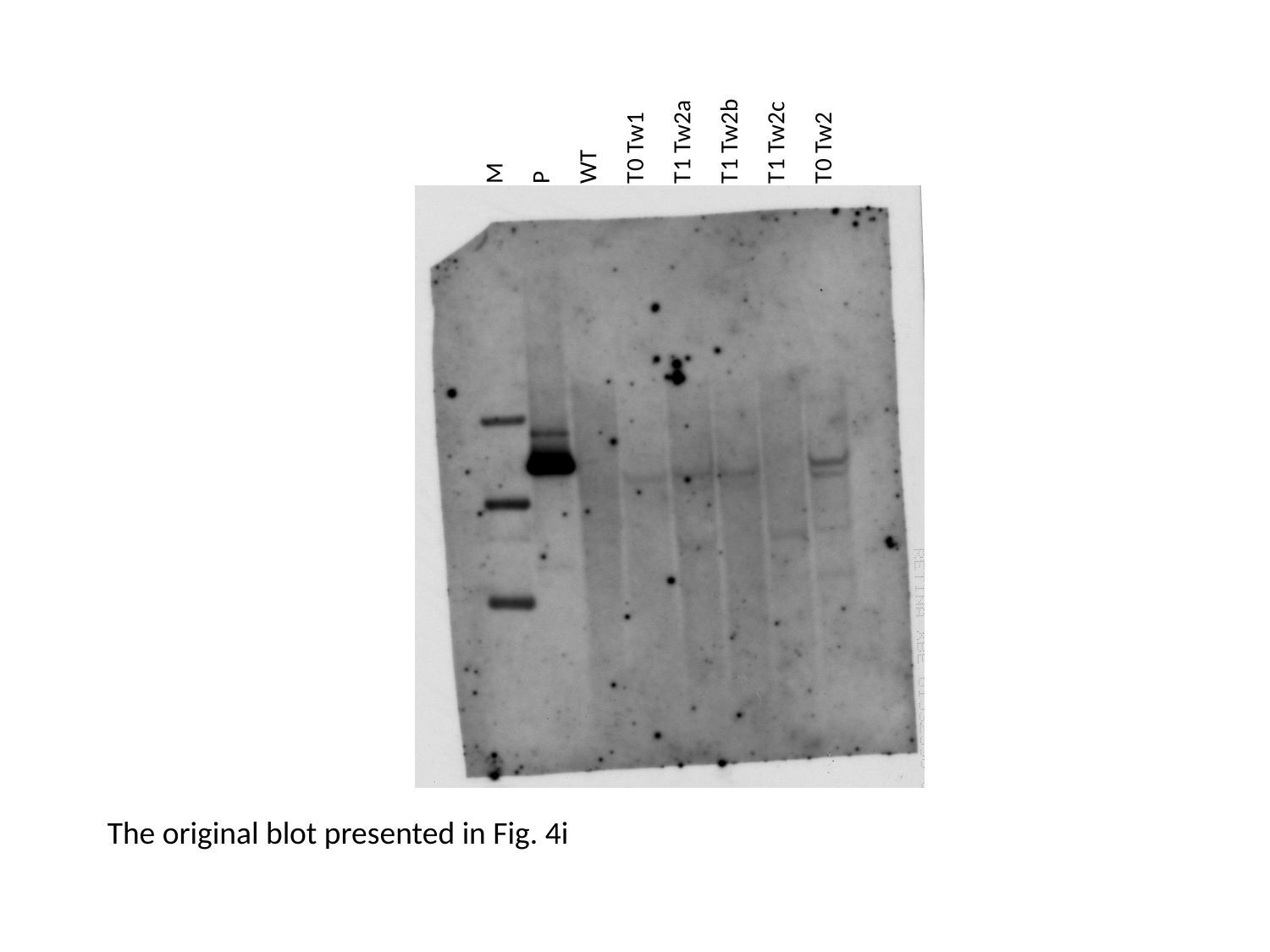

M
P
WT
T0 Tw1
T1 Tw2a
T1 Tw2b
T1 Tw2c
T0 Tw2
The original blot presented in Fig. 4i
