# Supplementary material for: A competence of embryo-derived tissues of tetraploid cultivated wheat species Triticum dicoccum and Triticum timopheevii for efficient and stable transgenesis mediated by particle inflow gun
Source: BMC Plant Biol. 2020 Oct 14;20(Suppl 1):442. doi: 10.1186/s12870-020-02580-4 (PMC7557024; doi:10.1186/s12870-020-02580-4)
Supplement: Supplementary file 3 — Additional file 3: Table S1. Segregation analysis of GFP expression in T1 progeny of transgenic plants of emmer wheat ‘Runo’. [file 12870_2020_2580_MOESM3_ESM.pdf]

Supplementary Material 3

A competence of embryo-derived tissues of tetraploid cultivated wheat species *Triticum dicoccum* and *Triticum timopheevii* for efficient and stable transgenesis mediated by particle inflow gun

Table S1 Segregation analysis of GFP expression in T_1 progeny of transgenic plants of emmer wheat 'Runo'

T0 plant	Number of T1 seeds tested	Endosperm					Seedlings				
		Observed GFP segregation ratio (positive:negative)	Ratio	χ^2 value for the expected segregation ratio (positive:negative)			Observed GFP segregation ratio (positive:negative)	Ratio	χ^2 value for the expected segregation ratio (positive:negative)		
				1:1	3:1	15:1			1:1	3:1	15:1
PI-1	75	37:38	1.0:1	0.01	26.35	252.52	60:15	4.0:1	27	1.00	24.20
PI-3	134	96:38	2.5:1	25.1	0.81	111.78	96:38	2.5:1	25.1	0.81	111.78
PI-4B	156	104:52	2.0:1	17.33	5.78	195.29	16:140	0.1:1	98.57	348.75	1856.01
PI-5B	234	198:36	5.5:1	112.15	11.54	33.32	164:70	2.3:1	37.76	3.01	223.65
PI-6	144	101:43	2.3:1	23.36	1.81	137.01	113:31	3.6:1	46.69	0.93	57.36
PI-7	130	104:26	4.0:1	46.8	1.73	41.95	104:26	4.0:1	46.8	1.73	41.95
PI-9	179	88:91	1.0:1	0.05	63.73	607.35	120:59	2.0:1	20.79	6.05	217.96
PI-10	152	106:46	2.3:1	23.68	2.25	149.59	106:46	2.3:1	23.68	2.25	149.59
PI-11	210	158:52	3.0:1	53.5	0.01	122.82	134:76	1.8:1	43.58	14.03	321.28
PI-12	89	62:27	2.3:1	13.76	1.35	88.13	53:36	1.5:1	3.24	11.33	177.65
PI-14	108	81:27	3.0:1	27	0.00	64.80	81:27	3.0:1	27	0.00	64.80
PI-15	200	84:116	0.7:1	5.12	116.16	914.11	0:200	0.0:1	200	600.00	3000.00
PI-16	240	167:73	2.3:1	36.82	3.76	239.22	171:69	2.5:1	43.55	1.80	207.36
PI-17	103	78:25	3.1:1	27.27	0.03	57.09	78:25	3.1:1	27.27	0.03	57.09
PI-18	146	118:28	4.2:1	55.48	2.64	41.65	118:28	4.2:1	55.48	2.64	41.65
PI-19	165	109:56	1.9:1	17.02	7.03	215.90	109:56	1.9:1	17.02	7.03	215.90
PI-20	147	147:0	Hom	147	49.00	9.80	147:0	Hom	147	49.00	9.80
PI-21	149	104:45	2.3:1	23.36	2.15	145.88	104:45	2.3:1	23.36	2.15	145.88

PI-23	146	91:55	1.7:1	8.88	12.50	246.01	105:41	2.6:1	28.05	0.74	118.77
PI-24	162	141:21	6.7:1	88.29	12.52	12.46	150:12	12.5:1	117.56	26.74	0.37
PI-25	126	60:66	0.9:1	0.29	50.38	457.62	41:85	0.5:1	15.37	121.15	805.69
PI-26	151	115:36	3.2:1	41.33	0.11	79.75	115:36	3.2:1	41.33	0.11	79.75
PI-27	138	71:67	1.1:1	0.12	40.82	421.43	59:79	0.7:1	2.89	76.53	612.50
PI-28	149	143:6	23.8:1	125.97	34.96	1.26	142:7	20.3:1	122.32	32.75	0.61
PI-29	360	179:181	1.0:1	0.01	122.68	1190.98	248:112	2.2:1	51.38	7.17	379.75
PI-30	190	113:77	1.5:1	6.82	24.43	380.97	14:176	0.1:1	138.13	463.50	2419.61
PI-31	119	81:38	2.1:1	15.54	3.05	133.96	81:38	2.1:1	15.54	3.05	133.96
PI-32B	178	121:57	2.1:1	23.01	4.68	201.78	113:65	1.7:1	12.94	12.59	278.29
PI-33	141	124:17	7.3:1	81.2	12.60	8.11	26:115	0.2:1	56.18	240.57	1364.82
PI-34	132	32:100	0.3:1	35.03	181.37	1088.40	0:132	0.0:1	132	396.00	1980.00
PI-35	133	133:0	Hom	133	44.33	8.87	131:2	65.5:1	125.12	39.16	5.11
PI-36	120	15:105	0.1:1	67.5	250.00	1352.00	5:115	0.0:1	100.83	321.11	1643.56
PI-37	304	217:87	2.5:1	55.59	2.12	259.59	218:86	2.5:1	5.02	1.75	252.01
PI-38	146	114:32	3.6:1	46.06	0.74	61.17	119:27	4.4:1	57.97	3.30	37.35
PI-39	111	42:69	0.6:1	6.57	81.76	592.22	62:49	1.3:1	1.52	21.70	272.03
PI-40	287	206:81	2.5:1	54.44	1.59	236.49	206:81	2.5:1	54.44	1.59	236.49
PI-41	36	14:22	0.6:1	1.78	25.04	184.92	16:20	0.8:1	0.44	17.93	149.36
PI-42	132	76:56	1.4:1	3.03	21.37	294.80	96:36	2.7:1	27.27	0.36	99.56
PI-43	129	71:58	1.2:1	1.31	27.41	329.92	62:67	0.9:1	0.19	49.93	459.56
PI-44	137	88:49	1.8:1	11.76	8.47	203.70	56:81	0.7:1	4.56	85.08	653.66
PI-46C	116	112:4	28.0:1	100.55	28.74	1.55	111:5	22.2:1	96.86	26.48	0.74
PI-48	156	141:15	9.4:1	101.77	19.69	3.02	144:12	12.0:1	111.69	24.92	0.55
PI-49	137	128:9	14.2:1	103.36	24.82	0.02	128:9	14.2:1	103.36	24.82	0.02
PI-50	155	107:48	2.2:1	22.46	2.94	161.62	4:151	0.0:1	139.41	433.55	2198.76
PI-51B	152	80:72	1.1:1	0.42	40.56	438.60	64:88	0.7:1	3.79	87.72	691.90
PI-52	136	101:35	2.9:1	32.05	0.04	88.13	100:36	2.8:1	30.12	0.16	94.90
PI-53	156	53:103	0.5:1	16.03	140.03	951.31	0:156	0.0:1	56	468.00	2340.00
PI-54	171	83:88	0.9:1	0.15	63.86	596.56	10:161	0.1:1	133.34	436.12	2254.98
PI-55	107	80:27	3.0:1	26.25	0.00	65.81	72:35	2.1:1	12.79	3.39	127.86

PI-56	149	114:35	3.3:1	41.89	0.18	75.58	124:25	5.0:1	65.78	5.37	28.19
PI-58	136	117:19	6.2:1	70.62	8.82	13.84	119:17	7.0:1	76.5	11.33	9.07
PI-59	142	112:30	3.7:1	47.35	1.14	53.64	111:31	3.6:1	45.07	0.76	58.83
PI-60	124	103:21	4.9:1	54.23	4.30	24.16	93:31	3.0:1	31	0.00	74.40
PI-61A	136	73:63	1.2:1	0.74	32.98	372.74	74:62	1.2:1	1.06	30.75	359.18
PI-62	115	74:41	1.8:1	9.47	6.96	169.67	74:41	1.8:1	9.47	6.96	169.67
PI-63	222	112:110	1.0:1	0.02	71.36	710.34	112:110	1.0:1	0.02	71.36	710.34
PI-64	169	130:39	3.3:1	49	0.33	81.67	130:39	3.3:1	49	0.33	81.67
PI-65	97	74:23	3.2:1	26.81	0.09	50.47	74:23	3.2:1	26.81	0.09	50.47
PI-66	131	78:53	1.5:1	4.77	16.69	261.62	78:53	1.5:1	4.77	16.69	261.62
PI-67	118	86:32	2.7:1	24.71	0.28	87.70	84:34	2.5:1	21.19	0.92	102.53
PI-68	139	71:68	1.0:1	0.06	42.42	431.94	71:68	1.0:1	0.06	42.42	431.94
PI-69	129	75:54	1.4:1	3.42	19.56	279.19	75:54	1.4:1	3.42	19.56	279.19
PI-70	128	78:50	1.6:1	6.13	13.50	235.20	13:115	0.1:1	81.28	287.04	1526.53
PI-71	154	65:89	0.7:1	3.73	88.32	698.23	74:80	0.9:1	0.23	59.65	548.86
PI-72	171	112:65	1.7:1	12.48	12.97	280.52	112:59	1.9:1	16.43	8.24	232.95
PI-73	101	45:56	0.8:1	1.2	49.93	417.18	0:101	0.0:1	101	303.00	1515.00
PI-78	40	10:30	0.3:1	10	53.33	322.67	10:30	0.3:1	10	53.33	322.67
PI-79	232	175:57	3.1:1	60.02	0.02	132.87	173:59	2.9:1	56.02	0.02	145.67
PI-81	260	224:36	6.2:1	135.94	17.25	25.60	243:17	14.3:1	196.45	47.26	0.04
PI-84	57	36:21	1.7:1	3.95	4.26	91.04	18:39	0.5:1	7.74	57.32	376.01
PI-85	145	111:34	3.3:1	40.89	0.19	73.20	111:34	3.3:1	40.89	0.19	73.20
PI-88	146	44:102	0.4:1	23.04	156.72	1008.31	10:136	0.1:1	108.74	361.65	1881.69
PI-90	151	89:62	1.4:1	4.83	20.77	312.27	27:124	0.2:1	62.31	262.75	1483.39
PI-91	159	154:5	30.8:1	139.63	40.51	2.62	154:5	30.8:1	139.63	40.51	2.62
PI-95	111	43:68	0.6:1	5.63	77.84	573.29	5:106	0.0:1	91.9	294.20	1508.84
PI-96	149	63:86	0.7:1	3.55	85.07	673.61	55:94	0.6:1	10.21	115.28	821.49
PI-97	125	73:52	1.4:1	3.53	18.37	266.59	0:125	0.0:1	125	375.00	1875.00
PI-98	301	196:105	1.9:1	27.51	15.68	421.18	162:139	1.2:1	1.76	72.01	819.03
PI-99	120	79:41	1.9:1	12.03	5.38	159.61	66:54	1.2:1	1.2	25.60	307.52
PI-103	138	45:93	0.5:1	16.7	132.26	880.43	4:134	0.0:1	122.46	382.62	1943.98

PI-106A	140	97:43	2.3:1	20.83	2.44	143.00	97:43	2.3:1	3.66	2.44	143.00
PI-109	263	198:65	3.0:1	67.26	0.01	153.04	216:47	4.6:1	108.6	7.13	60.61
PI-110	159	99:60	1.7:1	9.57	13.75	269.02	120:39	3.1:1	41.26	0.02	90.66
PI-111A	128	96:32	3.1:1	32	0.00	76.80	105:23	4.8:1	52.53	3.38	30.00
PI-113	158	60:98	0.6:1	9.14	115.52	838.86	74:84	0.9:1	0.63	66.84	593.50
PI-115	120	101:19	5.3:1	56.03	5.38	18.81	109:11	9.9:1	80.03	16.04	1.74
PI-116	258	93:165	0.6:1	20.09	208.79	1466.13	123:135	0.9:1	0.56	102.74	934.78
PM-1	46	37:9	4.1:1	17.04	0.72	13.92	34:12	2.8:1	10.52	0.03	30.89
PM-2	128	67:61	1.1:1	0.28	35.04	374.53	72:56	1.3:1	2	24.00	307.20
PM-4	107	76:31	2.5:1	18.90	0.90	94.28	3:104	0.0:1	95.36	297.45	1510.44
PM-6	42	32:10	3.2:1	11.52	0.03	22.10	33:9	3.7:1	13.70	0.29	16.51

if χ^2 value is above 3.84 ($P > 0.05$) the observed segregation ratio is not significantly different from the expected ratio
