# Supplementary material for: A competence of embryo-derived tissues of tetraploid cultivated wheat species Triticum dicoccum and Triticum timopheevii for efficient and stable transgenesis mediated by particle inflow gun
Source: BMC Plant Biol. 2020 Oct 14;20(Suppl 1):442. doi: 10.1186/s12870-020-02580-4 (PMC7557024; doi:10.1186/s12870-020-02580-4)
Supplement: Supplementary file 4 — Additional file 4: Figure S3. Molecular analysis of transgenic events of emmer wheat presented in Figure S1. The raw gels data. [file 12870_2020_2580_MOESM4_ESM.pptx]

## Slide 1
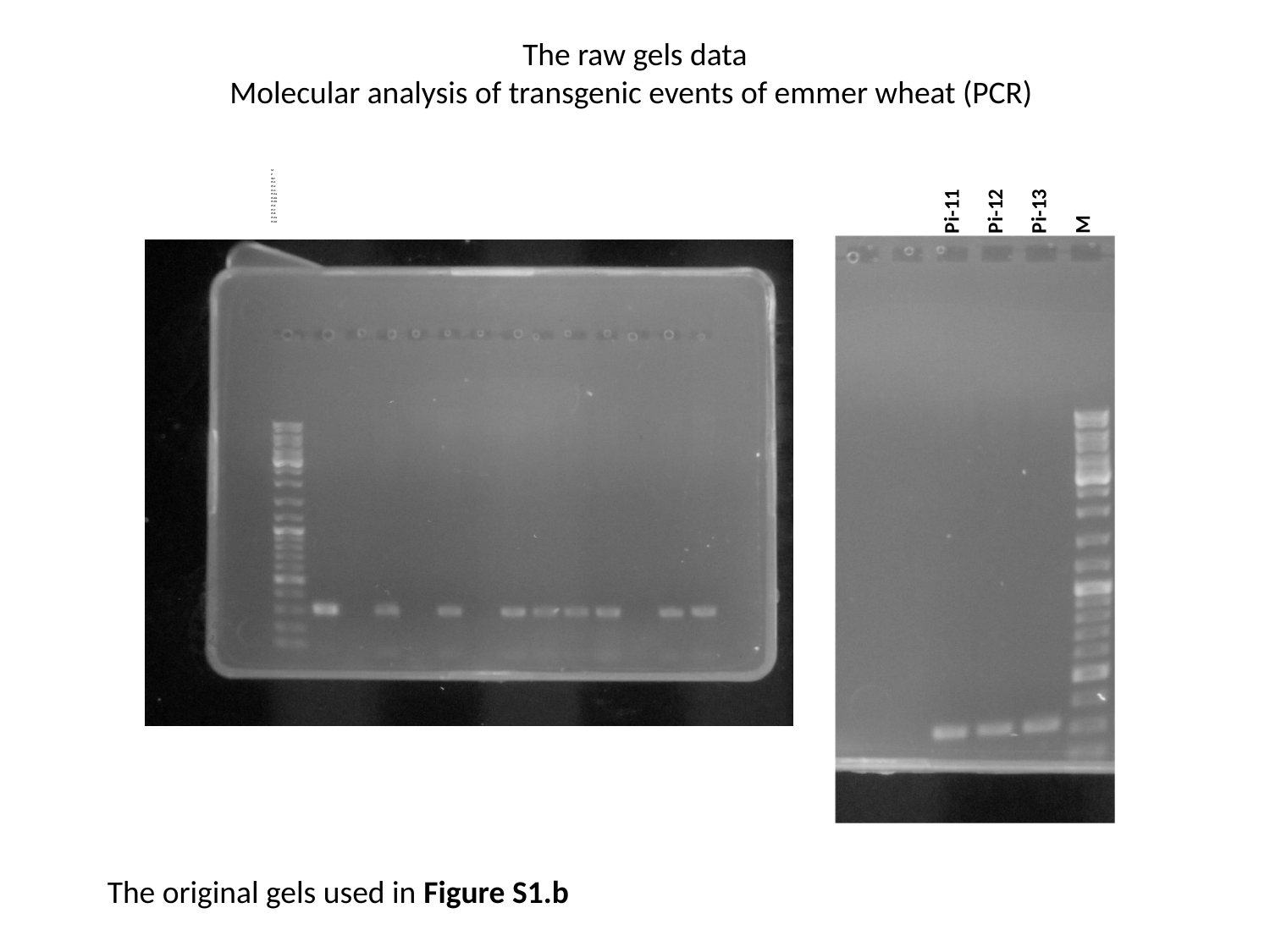

# The raw gels data Molecular analysis of transgenic events of emmer wheat (PCR)
M
P
WT
Pi-1
Pi-2
Pi-3
Pi-4a
Pi-4b
Pi-5b
Pi-6
Pi-7
Pi-8
Pi-9a
Pi-10
Pi-11
Pi-12
Pi-13
M
The original gels used in Figure S1.b

## Slide 2
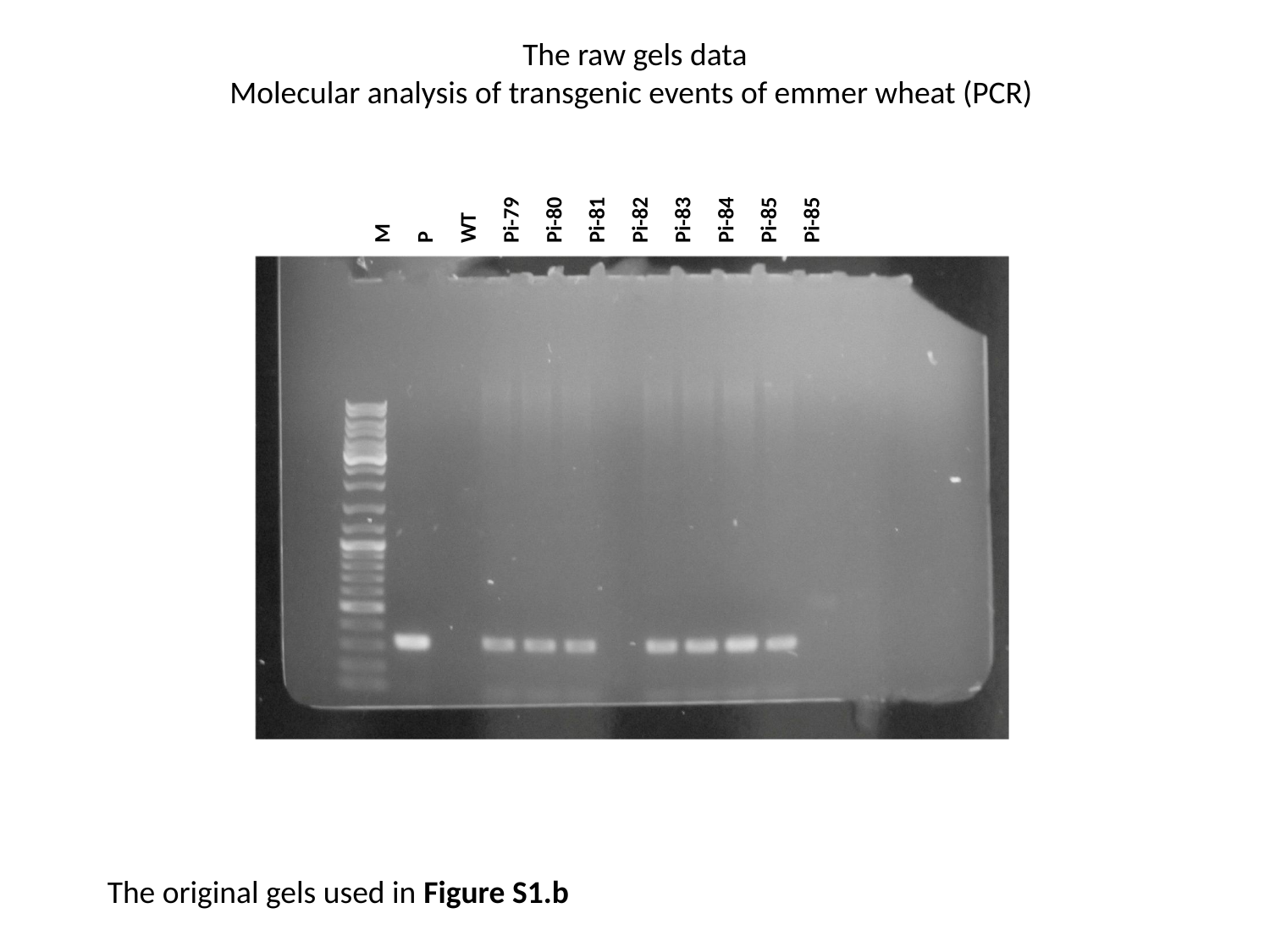

# The raw gels data Molecular analysis of transgenic events of emmer wheat (PCR)
M
P
WT
Pi-79
Pi-80
Pi-81
Pi-82
Pi-83
Pi-84
Pi-85
Pi-85
The original gels used in Figure S1.b

## Slide 3
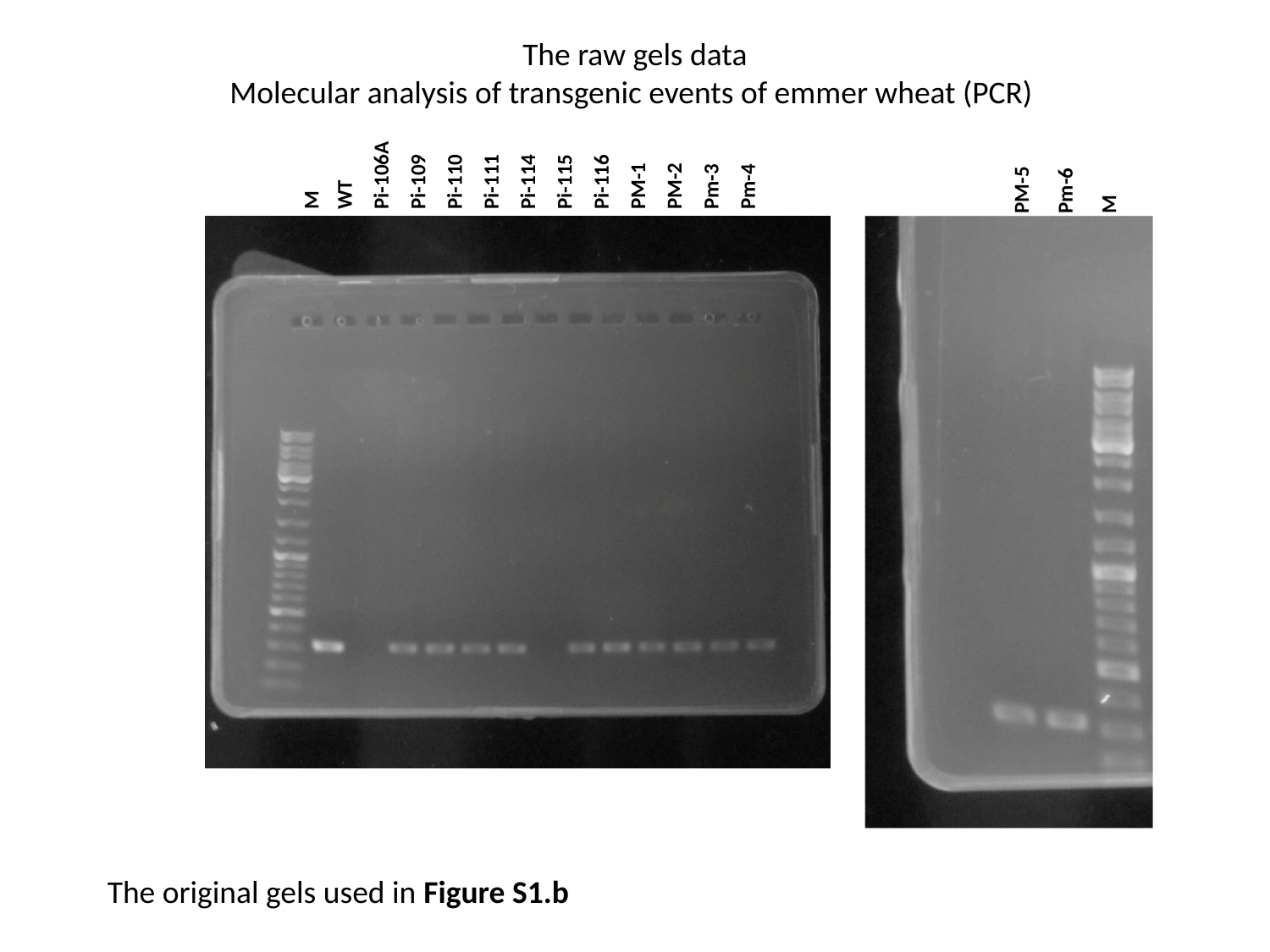

# The raw gels data Molecular analysis of transgenic events of emmer wheat (PCR)
M
WT
Pi-106A
Pi-109
Pi-110
Pi-111
Pi-114
Pi-115
Pi-116
PM-1
PM-2
Pm-3
Pm-4
PM-5
Pm-6
M
The original gels used in Figure S1.b

## Slide 4
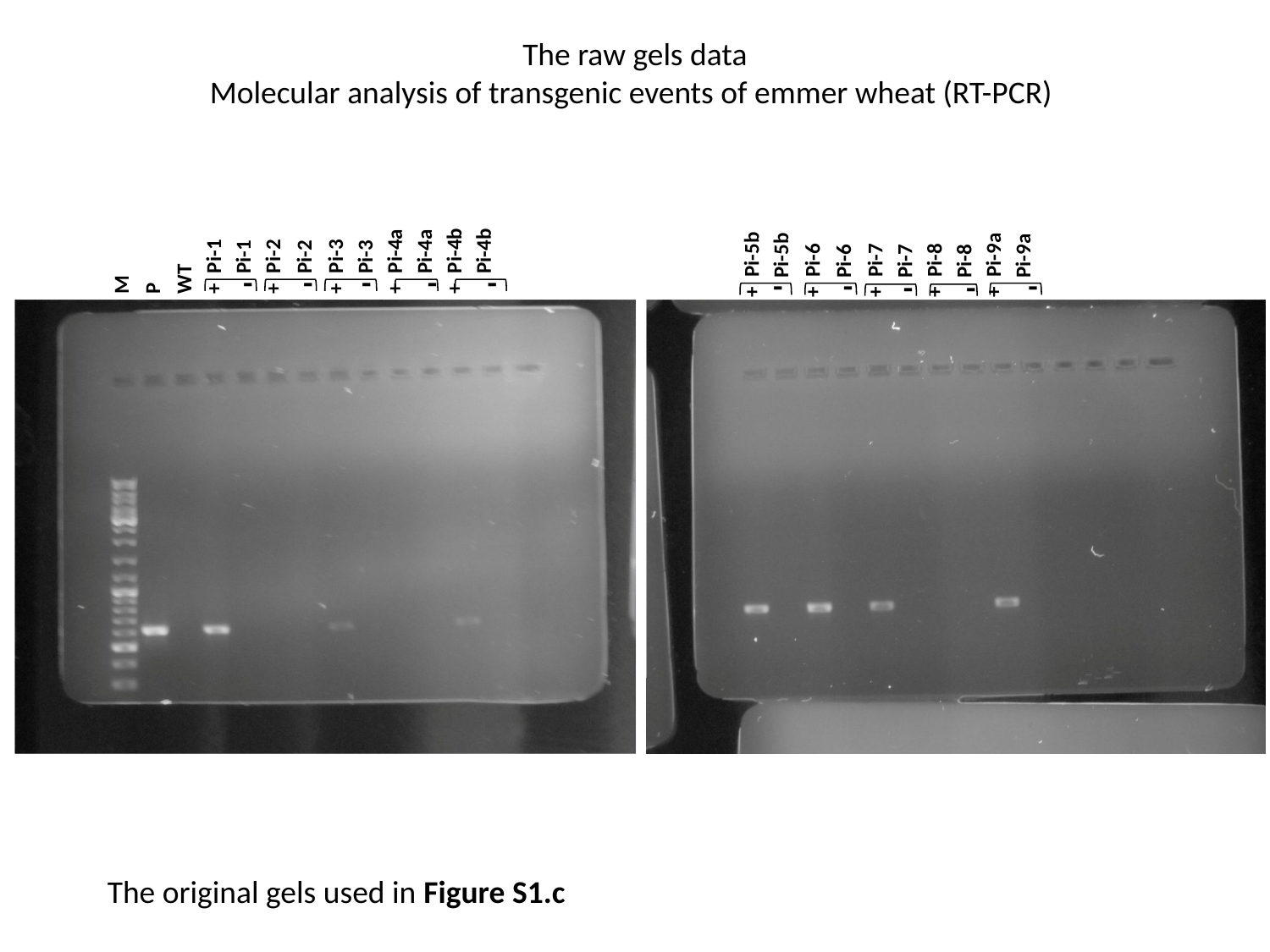

# The raw gels data Molecular analysis of transgenic events of emmer wheat (RT-PCR)
M
P
WT
+ Pi-1
 Pi-1
+ Pi-2
 Pi-2
+ Pi-3
 Pi-3
+ Pi-4a
 Pi-4a
+ Pi-4b
 Pi-4b
+ Pi-5b
 Pi-5b
+ Pi-6
 Pi-6
+ Pi-7
 Pi-7
+ Pi-8
 Pi-8
+ Pi-9a
 Pi-9a
-
-
-
-
-
-
-
-
-
-
The original gels used in Figure S1.c

## Slide 5
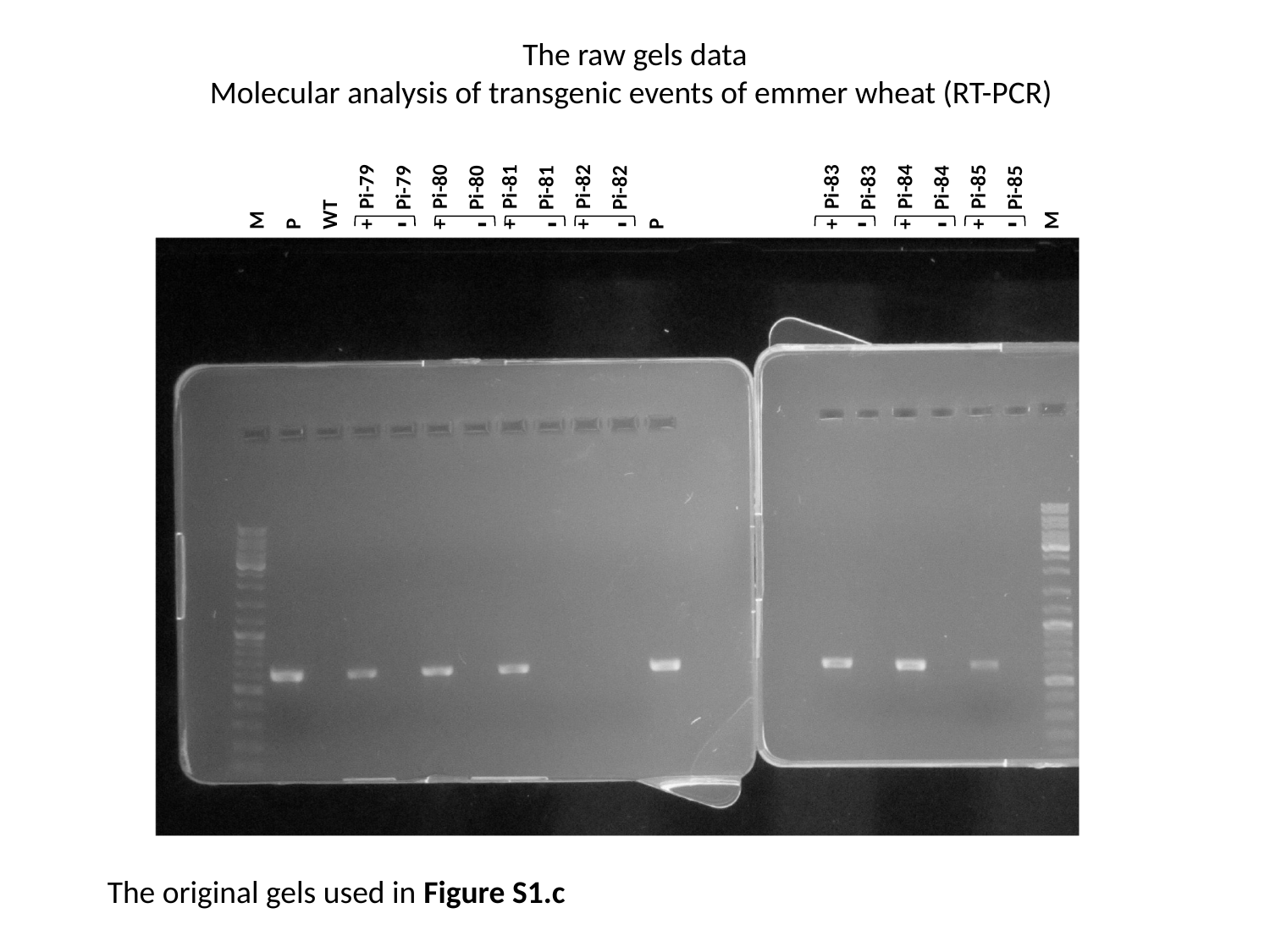

# The raw gels data Molecular analysis of transgenic events of emmer wheat (RT-PCR)
M
P
WT
+ Pi-79
 Pi-79
+ Pi-80
 Pi-80
+ Pi-81
 Pi-81
+ Pi-82
 Pi-82
P
+ Pi-83
 Pi-83
+ Pi-84
 Pi-84
+ Pi-85
 Pi-85
M
-
-
-
-
-
-
-
The original gels used in Figure S1.c

## Slide 6
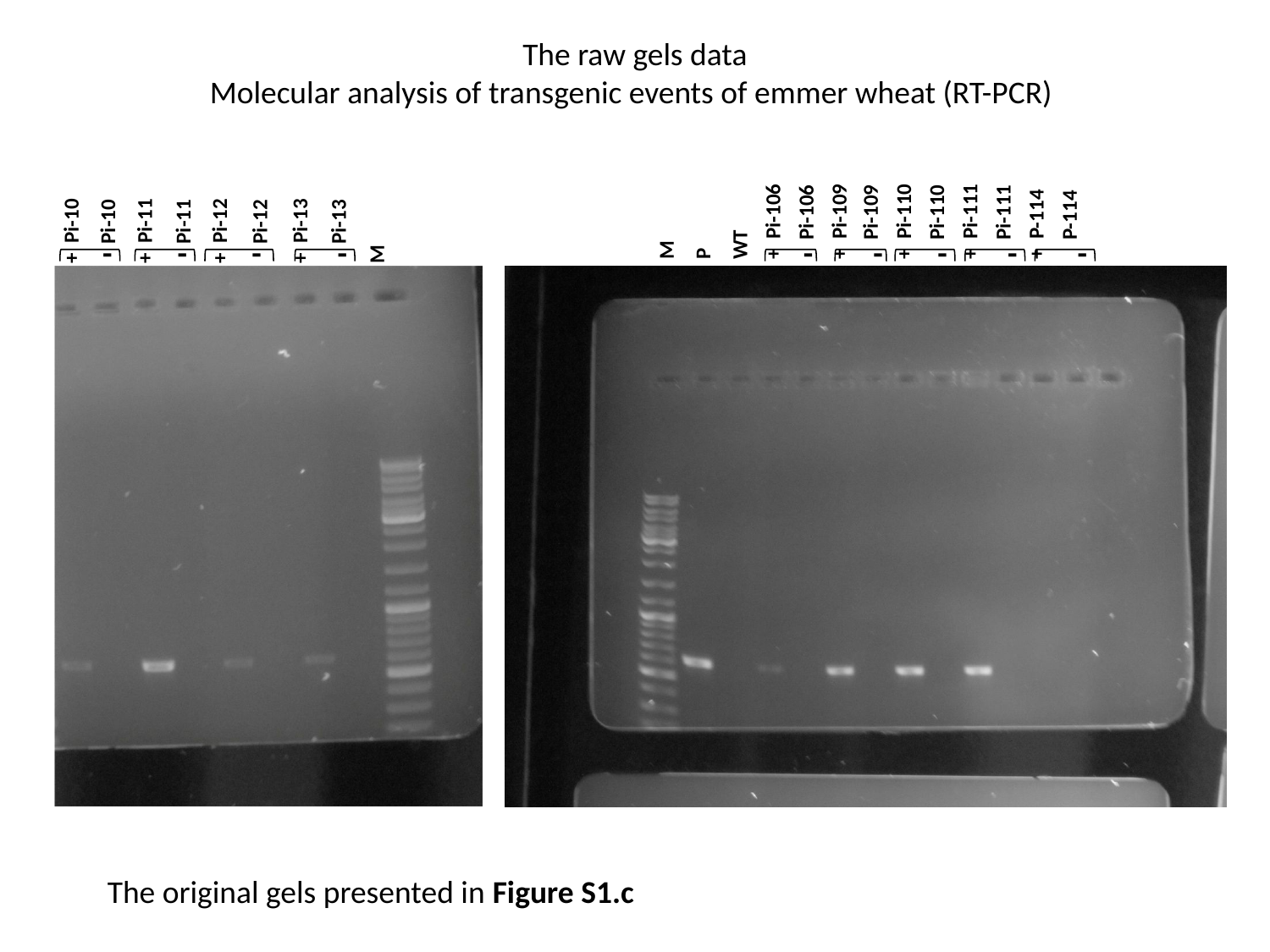

# The raw gels data Molecular analysis of transgenic events of emmer wheat (RT-PCR)
M
P
WT
+ Pi-106
 Pi-106
+ Pi-109
 Pi-109
+ Pi-110
 Pi-110
+ Pi-111
 Pi-111
+ P-114
 P-114
+ Pi-10
 Pi-10
+ Pi-11
 Pi-11
+ Pi-12
 Pi-12
+ Pi-13
 Pi-13
M
-
-
-
-
-
-
-
-
-
The original gels presented in Figure S1.c

## Slide 7
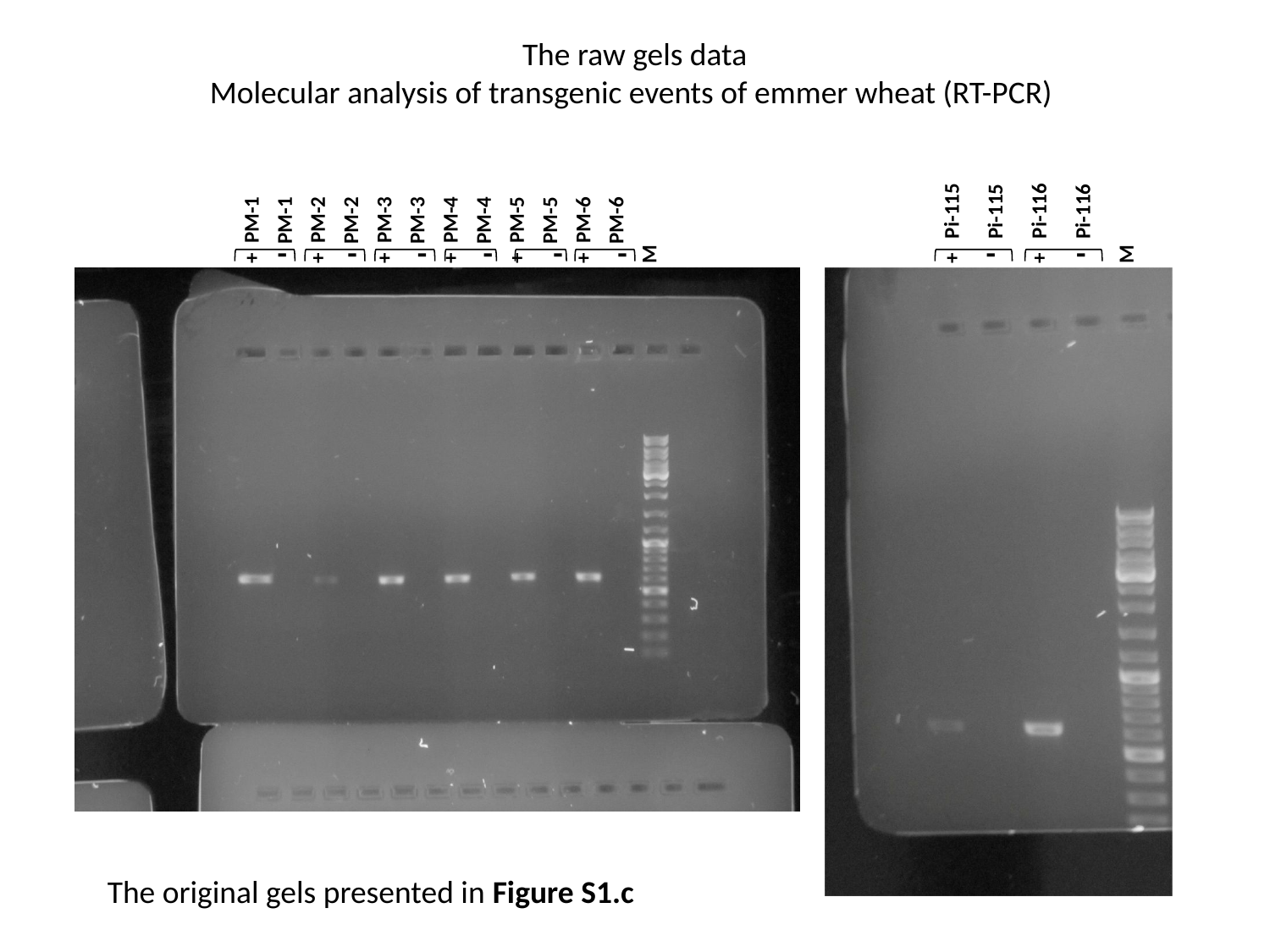

The raw gels data
Molecular analysis of transgenic events of emmer wheat (RT-PCR)
+ PM-1
 PM-1
+ PM-2
 PM-2
+ PM-3
 PM-3
+ PM-4
 PM-4
+ PM-5
 PM-5
+ PM-6
 PM-6
M
+ Pi-115
 Pi-115
+ Pi-116
 Pi-116
M
-
-
-
-
-
-
-
-
The original gels presented in Figure S1.c
